# Developing a prognostic stratification model based on glutathione metabolism in thyroid cancer and validating RRM2’s tumor−promoting role

**DOI:** 10.3389/fonc.2025.1700439

**Published:** 2025-11-17

**Authors:** Wei Ao, Teng-Hong Liu, De-Tao Yin, Wen-Xin Zhao

**Affiliations:** 1 Department of Thyroid Surgery, Fujian Medical University Union Hospital, Fuzhou, China; 2 Department of Thyroid Surgery, The First Affiliated Hospital of Zhengzhou University, Zhengzhou, China; 3 Fujian Clinical Research Center for Precision Management of Thyroid Cancers, Fujian Medical University Union Hospital, Fuzhou, China

**Keywords:** glutathione metabolism, thyroid cancer, *RRM2*, prognostic risk stratification modeling, LASSO-penalized cox regression

## Abstract

**Introduction:**

Glutathione (GSH), the most abundant antioxidant in cells, acts as free radical scavenger and detoxifying agent. Elevation of GSH metabolism protects tumor from damage of oxidant and even promotes tumor progression. However, the clinical value of GSH metabolism in thyroid cancer (THCA) remained largely unknown.

**Methods:**

The expression and prognostic value of GSH metabolism-related enzymes were first investigated using a large The Cancer Genome Atlas (TCGA) cohort of 510 THCA patients. To expand the prognostic application, a risk stratification model based on these enzymes was developed using the LASSO Cox regression algorithm. Patients were categorized into high- and low-risk groups based on the median risk score, and the model’s predictive performance for disease-freesurvival (DFS) was validated. Further correlation analysis, pan-cancer analysis (using TCGA and GTEx data), and detailed analysis across pathological types and TNM stages were performed to identify and characterize key molecules, such as RRM2. Finally, the biological role of *RRM2* was validated *in vitro* (CCK-8 and colony-formation assays) and *in vivo* (subcutaneous tumor formation in nude mice). Furthermore, the molecular mechanism underlying *RRM2*’s tumor-promoting function was preliminarily investigated through mRNA sequencing and subsequent experiments.

**Results:**

The majority of GSH metabolism–related enzymes were significantly upregulated in THCA tumor tissues and their expression was negatively associated with DFS. The LASSO Cox model stratified patients into high-risk and low-risk groups with significantly different DFS. High-risk status was also positively correlated with increased infiltration of naïve B cells, activated memory CD4+ T cells, helper T cells and regulatory T cells. *RRM2*, screened as a key molecule, exhibited high expression in THCA tissues, especially in more aggressive subtypes (classic and tall-cell variants of papillary THCA) and N stages. Paired-sample IHC confirmed higher *RRM2* in PTC versus adjacent tissue. High *RRM2* expression was significantly and negatively correlated with DFS. Functionally, *RRM2* overexpression promoted TPC-1 cell proliferation and colony formation (CCK-8 and colony assays) while knockdown suppressed growth. Subcutaneous tumor formation experiments recapitulated these findings. Mechanistically, *RRM2*’s oncogenic effects may be mediated through cell cycle regulation and activation of the PI3K/Akt signaling pathway.

**Discussion:**

GSH metabolism–related enzymes are upregulated in THCA and associate with a worse prognosis and an immune landscape suggestive of antigenic stimulation coupled with immunosuppression. *RRM2* is a tumor-promoting gene that correlates with aggressive clinicopathologic features and functionally drives thyroid tumor growth *in vitro* and *in vivo*. These data support further investigation of GSH metabolism and RRM2 as prognostic biomarkers and potential therapeutic targets in thyroid cancer.

## 1 Introduction

Thyroid cancer (THCA) is the most common malignant tumor of the endocrine system and consists of several histologic types, including papillary thyroid carcinoma (PTC), follicular thyroid carcinoma (FTC), medullary thyroid carcinoma (MTC), and Anaplastic Thyroid Carcinoma (ATC). Of these, PTC is the most common histologic subtype, accounting for 80-90% of all THCA case types (1). Currently, treatment decision for THCA mainly depends on clinicopathological factors like age, histologic type, tumor stage and so on, molecular risk stratification system is lacking (2). Although the traditional TNM staging system can provide a preliminary prognostic assessment, its limitations gradually emerge when facing the biological heterogeneity of tumors as well as the selection of individualized treatment strategies, making it difficult to comprehensively reflect the molecular characteristics and biological behaviors of tumors. In recent years, with the development of high-throughput sequencing technology, the construction of accurate prognostic models based on molecular markers has become an important research direction to improve the clinical management of THCA and guide treatment decisions.

Glutathione (GSH) is the most abundant antioxidant acting as free radical scavenger and detoxifying agent in almost all human cells (3). The biosynthesis of GSH including two important steps that happen in the cytosol. The first and limiting step is the conjugation of cysteine with glutamate to form the dipeptide γ-glutamylcysteine, which is catalyzed by γ-glutamyl-cysteine ligase (GCL). In the second reaction, glycine is added to the C-terminal of γ-glutamylcysteine catalyzed by glutathione synthetase (GSS). Under oxidative stress like reactive oxygen species (ROS), GSH is converted into oxidized state (GSSG) by GSH-dependent peroxidases, thus the GSH/GSSG ratio within cells reflects cellular oxidative stress and increased ratio indicates heavier oxidative stress (4).

Due to the high metabolic rate and/or the activation of ROS-coupled signaling pathways, oxidative stress always elevates in cancer cells (5, 6). Since intense oxidative stress leads to severe damage of biomolecules, triggering cell death, it is easily understood that GSH metabolism might be upregulated in tumor cells to protect them from damage of oxidant and even promote cancer cell proliferation and metastasis. For example, the nuclear factor erythroid 2-related factor 2 (NRF2), which controls the transcription of GCL, is stabilized and activated in breast cancer, promoting GSH biosynthesis and resistance to oxidative stress (7). In addition, NRF2 was reported to promote cancer cell proliferation by metabolic reprogramming and correlate with dismal survival outcomes in esophageal cancer, non-small cell lung cancer and pancreatic cancer (8–10). Prof. Martin O Bergo observed that N-acetylcysteine (NAC) increased lymph node metastases of malignant melanoma *in vivo* based on the GSH system (11). Furthermore, expression of GSH peroxidase and thioredoxin reductases were decreased in THCA tissue, indicating that the imbalance of the oxidant/antioxidant system played an important role in THCA (12). However, the clinical value of GSH metabolism in THCA remains ambiguous.

Based on large thyroid cancer cohort form The Cancer Genome Atlas (TCGA) database, we systematically analyzed molecular characteristics of 24 GSH metabolism-related enzymes and revealed two subgroups with distinct metabolic status and survival outcomes. To quantity the GSH metabolism status and stratify THCA patients, we further constructed a 9-gene based signature via LASSO-penalized Cox regression model. Furthermore, clinicopathological and microenvironment features were compared between the high- and low-risk patients. The robust and powerful metabolic index risk model could provide insightful suggestions to explore the molecular functions and mechanisms of GSH metabolism and might help guide clinical treatment decisions for THCA patients in future. Based on the THCA prognostic risk model, we screened the key molecule Ribonucleotide reductase M2 (*RRM2*) subunit, which has a potential pro-cancer role, to explore the functional role of *RRM2* and its molecular mechanism in PTC, and to investigate the effects of *RRM2* on the proliferation, apoptosis, migration invasion, cell cycle, and tumorigenicity of PTC cells *in vivo*.

## 2 Materials and methods

### 2.1 Data acquisition

TCGA RNA sequence level 3 normalized data and corresponding THCA clinical information were downloaded from UCSC Xena (https://xenabrowser.net/datapages/) for further analysis. The RNA-seq data of normal thyroid tissues were downloaded from the Genotype Tissue Expression (GTEx) database (http://commonfund.nih.gov/GTEx/). In addition, the study downloaded and integrated RRM2 RNA expression data for 30 different cancer types and their corresponding paracancerous tissues from the TCGA and GTEx databases.

### 2.2 Screening of the gsh metabolism related enzymes

We extracted enzymes that play a pivotal role in the GSH pathway and have the potential to be manipulated for the following analysis. We extracted 50 genes involved in the GSH metabolism according to Kyoto Encyclopedia of Genes and Genomes (KEGG) database, among these genes, 24 of them are annotated as enzymes based on the MetaCyc database. We further analyzed the functional role of these enzymes in THCA.

### 2.3 Bioinformatics analysis

Unsupervised hierarchical clustering of THCA samples was performed using R package “ConsensusClusterPlus v1.42.0”. The R package “survival v3.1-7” (https://cran.r-project.org/web/packages/survival/) was adopted to acquire the disease-free survival (DFS) through Kaplan-Meier estimation. We calculated the fold change and adjusted *p*-value using the DESeq2 v1.18.1 R package for all genes between different groups, and genes with an adjusted *p*-value less than 0.05 and |log2FoldChange| > 1 were considered as differentially expressed genes (DEGs). CIBERSORT v1.03 (13) was used to estimate the immune cell components in all samples. The nomogram was produced by R package “rms v2.4.1”.

### 2.4 Generation of a 9-gene signature-based model

To expand the application of the GSH metabolism related enzymes on THCA risk stratification in different datasets, we strived to establish a prognostic model to predict the THCA patient DFS status based on the expression level of these enzymes. We randomly selected 382 patients from the TCGA as the training dataset, and the remaining were treated as the validation dataset. The 24 putative GSH metabolism related enzymes were included to construct the model via LASSO Cox regression algorithm. The LASSO Cox regression algorithm was performed by using R package “glmnet v2.0-18” with default parameters. After 10-fold cross-validation by 1,000-time alteration, a risk signature including *RRM1, GSR, GCLM, IDH2, RRM2, ODC1, GGT5, OPLAH* and *GSTZ1* was finally adopted. And the regression coefficients were determined by the value of λ that gives minimum mean partial likelihood deviance. The risk score could be calculated as follows: Risk Score = k_1_×x_1_+k_2_×x_2_+…+k_i_×x_i_ (i = n). i represents for the selected enzyme, k for the regression coefficient and x for the log2(FPKM + 1) expression level. We further classified the samples into high- and low-risk group according to the median value of the risk scores.

### 2.5 Cell culture

PTC cell lines TPC-1(cat. no. 20240110-11) and IHH4(cat. no. 20240112-03) were purchased from iCell Bioscience Inc. For culture, all cells were incubated in RPMI 1640 medium (Gibco, Thermo Fisher Scientific) supplemented with 10% fetal bovine serum (TYCOTO; China Hanqiang (Guangzhou) Biotechnology Co.) Cells were kept in a humidified incubator (Thermo Fisher Scientific) at 37°C and 5% CO_2_.

### 2.6 Antibodies

Anti-Human/Mouse RRM2 (Cat.no. ab172476)/Anti-Human PI3K Kinase p85α+p55 Antibody (Cat.no. ab278545)/Goat anti-Rabbit IgG H&L (HRP, Cat. No. ab205718) and Goat anti-Mouse IgG H&L (HRP, Cat. No. ab97023) were Purchased from Abcam. Anti-Human Bax Antibody (Cat.no. 5023)/Anti-Human Bcl-2 Antibody (Cat.no. 3498)/Anti-Human MMP2 Antibody (Cat.no. 40994)/Anti-Human MMP9 Antibody (Cat.no. 13667)/Anti-Human N-Cadherin Antibody (Cat.no. 13116)/Anti-Human Vimentin Antibody (Cat.no. 5741)/Anti-Human Snail Antibody (Cat.no. 3879)/Anti-Human Akt Antibody (Cat.no. 4691)/Anti-Human Phospho-Akt Antibody (Cat.no. 4060)/Anti-Human PI3K Kinase p110α Antibody (Cat.no. 4249)/Anti-Human PTEN Antibody (Cat.no. 9188) were purchased from CST. Anti-Mouse PCNA (Cat.no. RMA-0145) and Anti-Mouse Ki-67 (Cat.no. RMA-0731) Antibodies were purchased from Maixin Bio-technology Development Company, Fuzhou, China. Anti-Human GAPDH Antibody (Cat.no. 10494)/Anti-Human Cyclin A2 Antibody (Cat.no. 18202)/Anti-Human Cyclin E2 Antibody (Cat.no. 11935)/Anti-Human CDK2 Antibody (Cat.no. 10122)/Anti-Human c-MYC Antibody (Cat.no. 10828)/Anti-Human Cyclin D1 Antibody (Cat.no. 60186) were purchased from Proteintech.

### 2.7 RNA extraction and RT-qPCR

In this study, cancer tissues and paired paracancerous thyroid epithelial tissues of 44 patients(age range 20–68 years) with PTC were collected by surgery from the Department of Thyroid Surgery, Union Hospital, Fujian Medical University (Fuzhou, Fujian, China). All diagnoses were confirmed by histopathological examination, and the patients’ clinicopathological characteristics were extracted from their medical records. Total RNA from THCA cell lines was extracted using the TRIzol reagents (Invitrogen; Thermo Fisher Scientific, Cat.no. 15596018). Subsequently, the prepared RNA was used to synthesize the first strand cDNA in accordance with the protocol of the All-In-One 5X RT MasterMix (abm, Cat.no. G592). Then, qPCR was performed in triplicate using the PerfectStart^®^ Green qPCR SuperMix (+Universal Passive Reference Dye) kit (Beijing TransGen Biotech Co,Cat.no. AQ602) according to the manufacturers’ protocol. The PCR protocol was conducted using the following conditions: 95°C for 30 sec, followed by 40 cycles of amplification (95°C for 5 sec and 60°C for 30 sec). All reactions were performed with a StepOnePlus Real-Time PCR System (Applied Biosystems, Thermo Fisher Scientific, Cat# 4376592). The specific primers for *RRM2* amplification were used as follows: Sense, 5′-TTGCCTGTGAAGCTCATTGG-3′ and antisense, 5′-CCTCTGATACTCGCCTACTCTC-3′. GAPDH primers were: Sense, 5′-GGTCGTATTGGGCGCCTGGTC-3′; Antisense, 5′-TGACGGTGCCATGGAATTTGCCA-3′. GAPDH was used as a reference control to normalize the transcriptional levels of target gene and data was calculated using the 2^−ΔΔCq^ method.

### 2.8 Immunohistochemistry and immunohistochemical score

Formalin−fixed, paraffin−embedded specimens were cut into 4 μm sections. Slides were deparaffinized in xylene, rehydrated through a graded ethanol series, and subjected to antigen retrieval in citrate buffer (pH 6.0) using microwave heating. Sections were incubated overnight at 4°C with the following primary antibodies: Anti-Human/Mouse *RRM2* (Cat. No. ab172476) — applied at 1:100 for human tissue sections and 1:1,000 for mouse tissue sections — and Anti-Mouse PCNA (Cat. No. RMA-0145) and Anti-Mouse Ki-67 (Cat. No. RMA-0731), which were used exclusively on mouse tissue sections (both PCNA and Ki-67 reagents were used as ready-to-use per the manufacturer’s instructions). After primary antibody incubation and washes, sections were processed with the Elivision™ Plus IHC detection system (ready-to-use; Cat. No. kit9903; Maixin Biotech, Fuzhou, China). Per the manufacturer’s protocol, slides were incubated with the kit amplifier/enhancer for 20 min at room temperature (≈25°C), followed by incubation with the HRP-conjugated secondary reagent for 30 min at room temperature. Immunoreactivity was visualized with 3,3′-diaminobenzidine (DAB). Following DAB development, nuclei were counterstained with hematoxylin, differentiated and blued in ammonia water, and coverslips were mounted with neutral resin.

Immunostained slides were independently reviewed by senior pathologists (≥5 years’ experience) at the Department of Pathology, Fujian Medical University Union Hospital, who were blinded to all clinical information. Staining was scored semi−quantitatively based on the percentage of positive cells (0 = 0%; 1 = 1–24%; 2 = 25–49%; 3 = 50–74%; 4 = 75–100%) and staining intensity (0 = negative; 1 = weak; 2 = moderate; 3 = strong). The final H−score was obtained by multiplying the proportion score by the intensity score (14, 15).

### 2.9 Western blotting

Total proteins were isolated from cells using a lysate prepared in the ratio of RIPA: protease inhibitor: phosphatase inhibitor: PMSF = 100:1:1:1. Then the amount of protein was quantity using Enhanced BCA Protein Assay Kit (BOSTER Biological Technology co, Cat. no. AR0197A). Equal amounts of protein (20 μg) were separated by 8%~15% sodium dodecyl sulfate-polyacrylamide gel electrophoresis and then transferred to polyvinylidene difluoride membranes (Millipore, Cat.no. IPVH00010). Prior to primary antibody incubation, membranes were blocked with a protein-free rapid blocking solution (Cat. No. AR0041; Wuhan Boster Biological Technology Co., Ltd.) for 20 min at room temperature on a shaker (50–60 rpm). Following blocking, membranes were incubated with primary antibodies overnight at 4°C. The anti-GAPDH antibody (Anti-Human GAPDH, Cat. No. 10494; Proteintech) was used as the internal loading control at a working dilution of 1:6,000. After three washes with TBST, membranes were incubated with HRP-conjugated secondary antibodies—Goat anti-Rabbit IgG H&L (HRP, Cat. No. ab205718) and Goat anti-Mouse IgG H&L (HRP, Cat. No. ab97023) (Abcam)—each at a 1:6,000 dilution for 1 h at room temperature (20–25°C) on a shaker. The immunostained proteins were visualized with Efficient chemiluminescence kit (Beijing Dingguo Changsheng Biotech Co, Cat. no. GE2301). Image J software (National Institutes of Health, USA; open-source software available at https://imagej.nih.gov/ij/) was used to examine the gray values of each primary antibody and glyceraldehyde 3-phosphate dehydrogenase (GAPDH).

### 2.10 Overexpression and knockdown of RRM2 gene in TPC-1 and IHH4 cells

For upregulating *RRM2* expression in cells, we obtained a full-length *RRM2* sequence fragment using a PCR method and subsequentlycloned it into vector LV18, verified by DNA sequencing and transfected 293T cells (see **Supplementary Materials** for details). The collected lentiviruses were used to infect TPC-1 and IHH4 cells and the cells were screened with cell cultures containing 3 ug/mL puromycin. Finally, TPC-1 and IHH4 cells were collected for western blotting to assess the efficiency of *RRM2* overexpression.

To knock down *RRM2* protein expression, *RRM2* shRNA was developed to silence the *RRM2* gene. Lenti-*RRM2* shRNA vector was constructed using *RRM2* shRNA oligonucleotides with LV 2N (U6/Puro) vector. Based on the manufacturer’s instructions (GenePharma, Shanghai, China), lentiviral (Lenti-*RRM2* shRNA or Lenti-shRNA) (MOI = 15) was applied to transfect TPC-1 and IHH4 cells with 5 μg/ml polybrene(Sigma, Cat# H9268) for 12 h. After selection of 3 μg/ml puromycin(Beyotime, Cat# ST551), TPC-1 and IHH4 cells were harvested for western blotting to assess *RRM2* knockdown efficiency.

### 2.11 Cck-8 assay

Cells (1000 per well) were incubated into 96 well and cell vitality was assessed by Cell Counting Kit-8 (Dojindo, Cat.no. CK04) at 24, 48, 72 and 96 h according to the manufacturer’s instructions. Absorbance was recorded at 450 nm with a multifunctional enzyme marker (BioTek Instruments Inc., USA).

### 2.12 Colony-formation analysis

Cells (1000 per well) were plated in culture plates for 1 weeks at 37°C in a humidified environment with 5% CO_2_ and stained with crystal violet staining solution(1%) for 10~20 min. The stained colonies were imaged using a camera and counted using a microscope.

### 2.13 Subcutaneous tumor formation in nude mice

Tumor-bearing mice were monitored daily, and humane endpoints were strictly applied. Humane endpoints: Animals were euthanized if tumor volume exceeded 2000 mm³, if tumors reached 10% of body weight, if body weight loss exceeded 20–25%, or if signs of cachexia or severe distress were observed. Euthanasia was performed under deep anesthesia induced by intraperitoneal injection of 1% pentobarbital sodium at 0.1–0.2 mL per 10 g body weight (≈100–200 mg/kg); deep anesthesia was confirmed by flaccidity and the absence of a pain response to a hind-limb pinch, after which cervical dislocation was carried out. All procedures were carried out in accordance with Fujian Medical University animal care and welfare guidelines and institutional animal welfare regulations, and were approved by the Institutional Animal Care and Use Committee of Fujian Medical University (Approval No. FJMU20240245).

Eighteen 4-5-week-old female BALB/c nude mice were purchased and housed in an animal house (SPF grade), divided into 3 groups of 6 mice each, which were the TPC-1 cell-negative control group (n=6), the experimental group injected with *RRM2*-KD cells (n=6), and the experimental group injected with *RRM2*-OE cells (n=6). The mouse cages were prepared in advance, washed with soapy water, sprayed with 1% peroxyacetic acid on the inside, sealed and sterilized for 24 h, and then ventilated and dried. Inside the cages were placed sterile UV-sterilized mouse food, water and bedding, and 6 nude mice were kept in each cage. The animal room simulated daylight for 12 h day and night, and the condition of the mice was observed every day, and the bedding was changed and the food and water were added in a timely manner. When the cells were expanded to ~75–90% confluence, the cells were washed twice with PBS to remove the residual serum, digested and centrifuged and resuspended with sterile PBS, the cells were counted and the cell concentration was adjusted to 1×10^7^/ml, and the cell suspension was stored in EP tubes and kept on ice. The nude mice were randomly divided into 3 groups, and each group was marked with an ear piercing to distinguish them from the others. 200 μl of cell suspension containing 2 × 10^6^ cells was inoculated subcutaneously on the right axillary region of the nude mice in each of the 3 groups, and the wounds were gently pressed with a sterile cotton ball to prevent the inoculum from flowing out after the inoculation. After inoculation, the nude mice were put back into the cage without any special discomfort. BALB/c nude mice (4–5 weeks old) were inoculated subcutaneously; tumor measurements with caliper began on day 10 post-inoculation and were thereafter recorded every three days. Tumor length (long diameter) and width (short diameter) were recorded with sterile vernier calipers. When the test was stopped, the daily change in the volume size of the tumor was calculated, and the formula for calculating the volume of the tumor was V=long diameter × long diameter × short diameter/2. Experiments were terminated on day 19 post-inoculation, at which point tumors had reached sizes considered sufficient for downstream assays (histology, protein/RNA extraction, and functional analyses). Animals were euthanized and then cervical vertebrae were dislocated under confirmed deep anesthesia, and tumors were harvested for further study.

### 2.14 Cell cycle analysis

Cells (2 × 10^5^) were seeded in 6-well plates and cultured for 24h. Cells were digested and collected in a new EP tube and fixed them with cold ethanol at 4°C overnight. After this, 500 µl propidium iodide (PI) and RNase A (Elabscience, Cat. no. E-CK-A351) (1:9) were applied to incubate cells in the darkness. Approximately 1.5 × 10^5^ cells per well were acquired for analysis. The results were analyzed using a Flow Cytometry System (C6 plus). The percentage of different cell cycles was calculated using FlowJo v7.6.1 (BD Life Sciences, licensed copy distributed via Fujian Medical University; local download: https://ggjszx.fjmu.edu.cn/news/systemview?id=244).

### 2.15 Cell apoptosis assay

Cell apoptosis was assessed by APC-Annexin V Binding Apoptosis Assay Kit (Elabscience, Cat.no. E-CK-A218) following the manufacturer’s protocols. Cells were washed re-suspended in binding buffer containing propidium iodide (PI) and Annexin V-APC. Approximately 3 × 10^4^ cells per well were acquired for analysis. Stained cells were analyzed by Flow Cytometry System (C6 plus).

### 2.16 Wound-healing assay

Cells (6× 10^5^) were seeded in 6-well plates and cultured to >90% confluence. Wounds were scratched using a 200-μl plastic pipette tip. After PBS wash 2~3 times, cells were maintained in serum-free RPMI 1640 medium (Gibco, Thermo Fisher Scientific) for the duration of the assay. Wounded areas were photographed by phase-contrast microscopy at 24 h and 48 h, respectively.

### 2.17 Transwell assay

Cells were suspended in serum-free medium, counted, and the cell concentration adjusted to 2.5 × 10^5^ cells/mL. A volume of 200 µL of this suspension (containing 5.0 × 10^4^ cells) was then seeded into the upper chamber with coated Matrigel (Corning,Cat.no. 354234) of a 24-well chamber (Corning, Cat.no. 3422). The 24-well chamber was fitted with polycarbonate membranes (6.5 mm diameter, 0.33 cm² growth area) containing 8.0 µm pores. Six-hundred-microliter media containing 10% FBS were added to the lower chamber overnight. Migrated cells were fixed with 4% paraformaldehyde and stained with crystal violet. Cells were rinsed and counted from random fields. All rights reserved (×100 magnification). Each experiment was conducted triplicate.

### 2.18 RNA sequencing and transcriptome analysis

#### 2.18.1 RNA extraction and library preparation

Total RNA was extracted from cells using TRIzol reagent (Invitrogen, Carlsbad, CA) according to the manufacturer’s instructions. RNA integrity was evaluated on an Agilent 2100 Bioanalyzer (Agilent Technologies, Santa Clara, CA). Indexed, directional RNA-seq libraries were constructed with the NEBNext^®^ Ultra™ Directional RNA Library Prep Kit for Illumina (New England Biolabs, Ipswich, MA). Briefly, poly(A)+ mRNA was isolated using oligo(dT) beads, fragmented, and reverse transcribed into first-strand cDNA. Second−strand synthesis, end repair, adaptor ligation, and PCR amplification were performed to generate the final sequencing libraries. Libraries were sequenced on an Illumina NovaSeq 6000 platform (Novogene Co., Ltd., Beijing, China) using a paired−end 150 bp (PE150) strategy.

#### 2.18.2 Data quality control

Raw sequencing reads were processed with fastp to remove adapters and low−quality bases, yielding high−quality “clean” reads. The cleaned data were assessed for base quality (Q20, Q30) and GC content. All downstream analyses were based on these filtered reads.

#### 2.18.3 Alignment to the reference genome

The latest reference genome assembly and corresponding gene annotation files were downloaded from the genomic database. A HISAT2 v2.0.5 index was built for the reference genome, and paired−end clean reads were aligned using HISAT2 v2.0.5 with default parameters.

#### 2.18.4 Quantification of gene expression

Mapped reads were counted against gene features using FeatureCounts (v1.5.0−p3). Gene expression levels were normalized to fragments per kilobase of transcript per million mapped reads (FPKM) by dividing the read counts by the gene length and sequencing depth.

#### 2.18.5 Differential expression analysis

Each cell line was assayed in biological triplicate. Differential expression between two conditions was determined using DESeq2 (v1.20.0). P values were adjusted for multiple testing using the Benjamini–Hochberg procedure to control the false discovery rate (FDR). Genes with an adjusted P ≤ 0.05 were considered significantly differentially expressed.

#### 2.18.6 Functional enrichment analysis

Gene Ontology (GO) and Kyoto Encyclopedia of Genes and Genomes (KEGG) pathway enrichment analyses of differentially expressed genes were performed using ClusterProfiler (v3.8.1). Terms with an adjusted P < 0.05 were deemed significantly enriched.

### 2.19 Statistics

R v4.2.1 (The R Foundation for Statistical Computing; available at https://cran.r-project.org/) was used for all statistical analyses. To assess whether datasets follow a Gaussian distribution, the Shapiro–Wilk normality test was performed. If the data were Gaussian, parametric tests were performed (two-tailed unpaired t-tests). If the data were non-Gaussian, nonparametric tests were applied (Wilcoxon rank test or Spearman correlation). The results were considered statistically significant when P < 0.05, or a lower threshold when indicated by the appropriate test. Survival analysis was performed using the Kaplan-Meier method. A log-rank test was used to evaluate the significance of the difference between different Kaplan-Meier curves. The hazard ratio was determined using a Cox proportional hazards model. The test used and the statistical significance are reported in each figure and table.

Unless otherwise stated, data are presented as mean ± standard deviation (SD). The number and type of replicates for each experiment are as follows: RT-qPCR validation — n = 4 biological replicates for TPC-1 cells, n = 3 biological replicates for IHH4 cells and clinical tissue. Western blot — n = 4 biological replicates. CCK-8 assays — n = 5 technical replicates (wells) per group. Colony-formation assays — n = 3 biological replicates (independent experiments). Wound-healing and Transwell invasion assays — n = 3 technical replicates per condition. Flow-cytometric assays for cell-cycle distribution and apoptosis — n = 3 biological replicates.

## 3 Results

### 3.1 GSH metabolism related enzymes highly expressed in THCA

**Figure 1:**
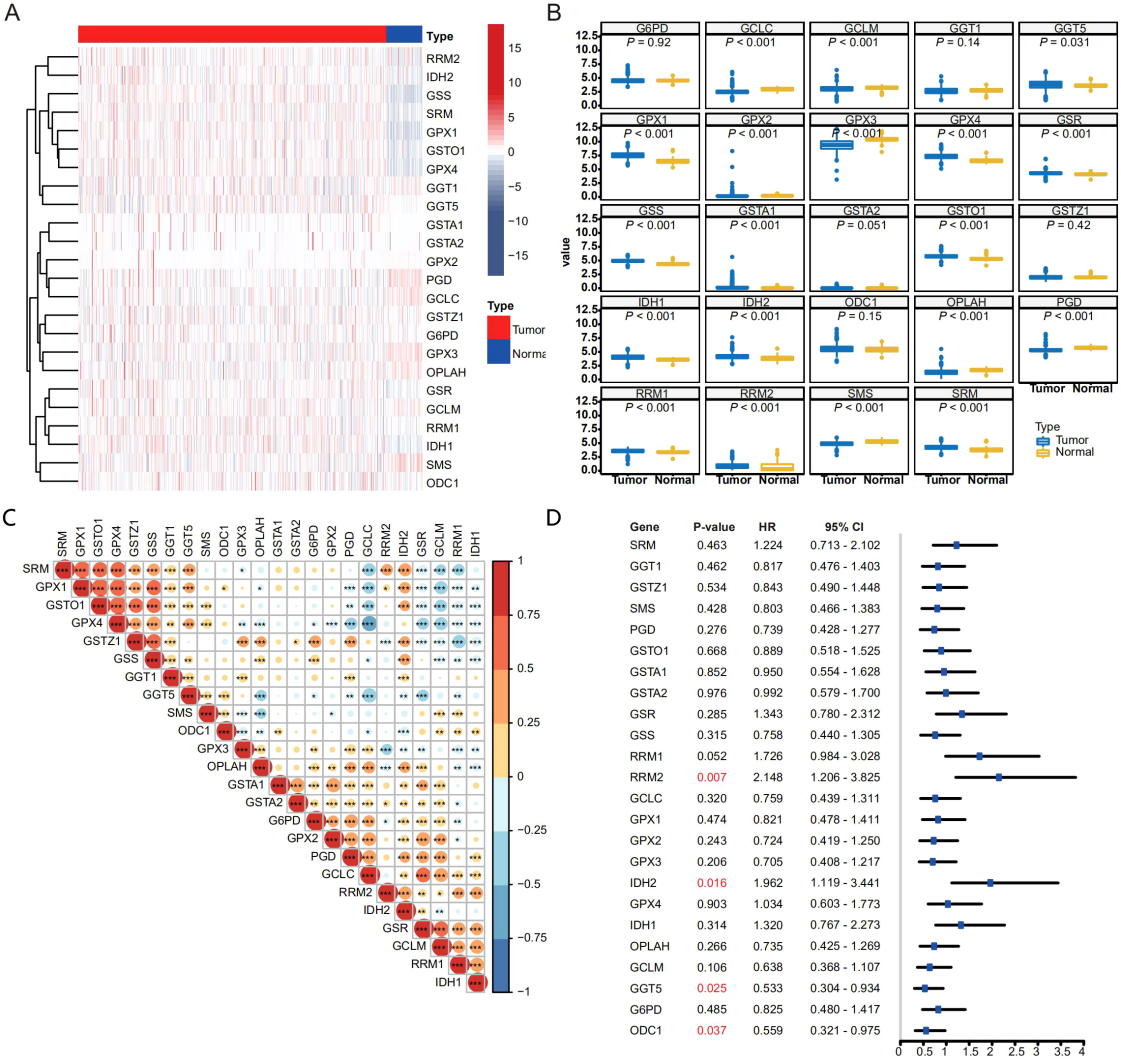
Expression pattern and prognostic relevance of GSH metabolism–related enzymes in THCA. **(A, B)** Heatmap **(A)** and box plot **(B)** showing the expression pattern of GSH metabolism related enzymes between THCA and normal thyroid samples. For **(B)**, gene expression values were log_2_-transformed [log_2_(TPM + 1)] prior to analysis. For each gene, differences between tumor and normal samples were tested using two-sided Welch’s t-tests (unpaired, not assuming equal variances). P-values across the multiple gene/tissue comparisons were adjusted using the Holm procedure. **(C)** Spearman correlation matrix of GSH metabolism–related enzyme expression across the THCA cohort (*p < 0.05, **p < 0.01, and ***p < 0.001). **(D)** Forest plot depicting hazard ratios (with 95% confidence intervals) for each GSH metabolism–related enzyme in univariate survival analysis.

In order to decipher the role of GSH metabolism related enzymes in THCA, we collected expression data of 510 THCA tumor samples from the TCGA and 58 normal thyroid samples from the GTEx database. As supposed, most GSH metabolism related enzymes like *GGT5*, *GPX1*, *GPX2*, *GPX4*, *GSR*, *GSS*, *GSTA1*, *GSTA2*, *GSTO1*, *IDH*, *IDH2*, *RRM1*, *RRM2* and *SRM* were highly expressed in THCA samples, while expression of *GCLC*, *GCLM*, *GPX3*, *OPLAH*, *PGD* and *SMS* were decreased in tumor tissues (**Figures 1A, B**). The correlation heatmap of 24 genes is shown in the **Figure 1C**. The significantly increased expression of the GSH metabolism related enzymes demonstrated that it might play an essential role in progression of THCA and might be able to predict the survival outcomes of THCA patients. We wonder whether the expression of GSH metabolism related enzymes could predict the progression of THCA patients. Calculating the Cox proportional hazard ratios (HRs), we found that high expression of *RRM2* and *IDH2* were negative prognostic factors of DFS, while high expression of *GGT5* and *ODC1* were correlated with better DFS (**Figure 1D**).

### 3.2 Establishment of a 9-gene based risk stratification model

**Figure 2:**
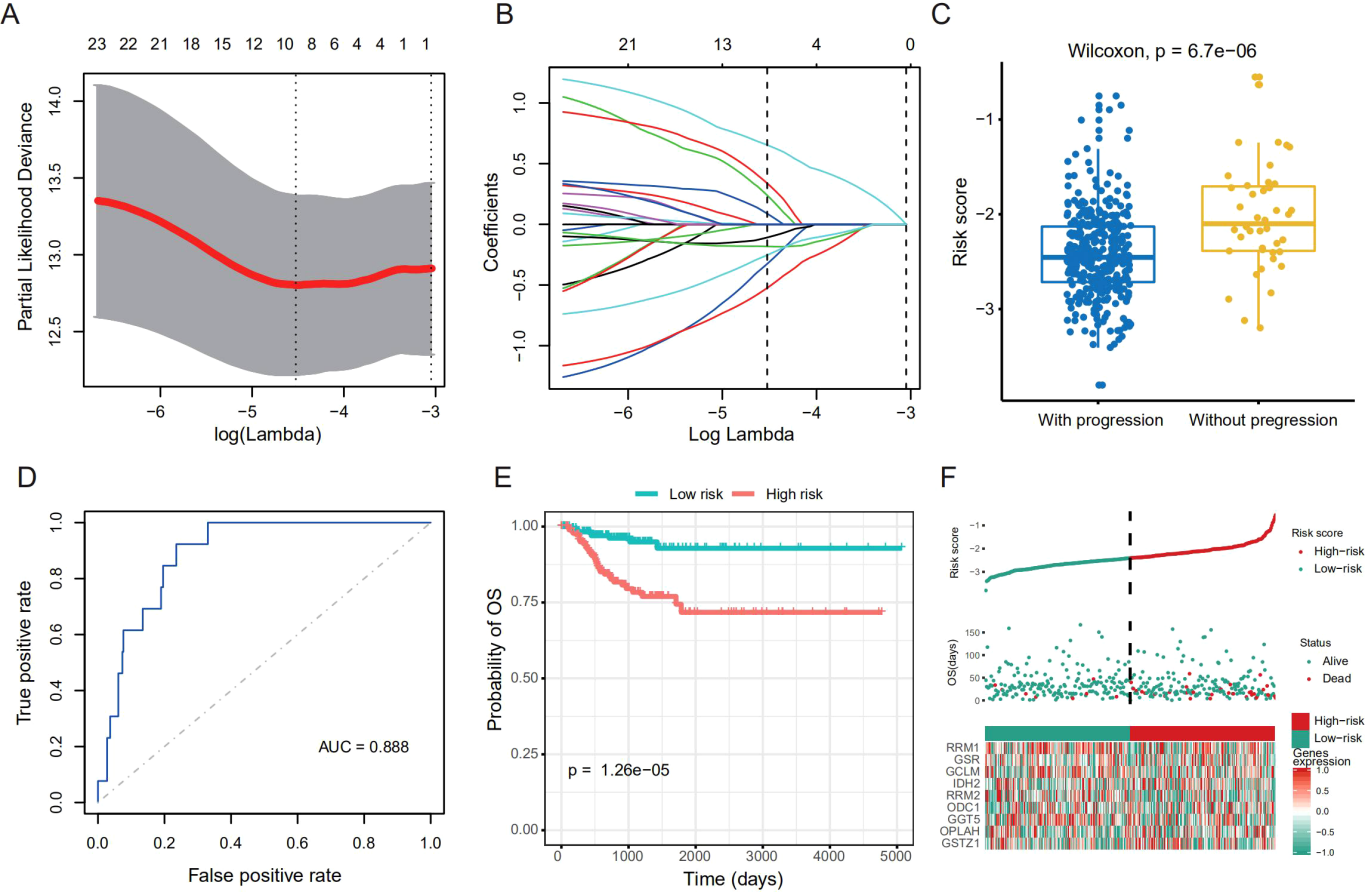
Establishment of risk stratification model based on the GSH metabolism related enzymes. **(A)** Tuning parameter (lambda) selection in the LASSO model. **(B)** Coefficient profile plot was produced against the log lambda sequence. **(C)** Boxplot shows difference of the risk score between patients with progression and those without in the training dataset. We used the Wilcoxon rank-sum test (Mann–Whitney U, two-sided) to compare the two independent groups. **(D)** Receiver operating characteristic (ROC) curves show the predictive efficiency of the risk stratification model in the training dataset. **(E)** Kaplan–Meier curves of DFS for the high- and low-risk patients in the training dataset. **(F)** Risk plot for the THCA patients in the training dataset. Each panel consists of three rows: top row showed the risk score distribution for the high- and low-risk score group; middle row represents the THCA patients’ distribution and DFS status; the bottom row presents heatmap of expression of the 9-prognostic metabolism-related enzymes.

To further expand the application of GSH metabolism related enzymes on THCA risk stratification in different datasets, we strived to establish a prognostic model to quantify the expression levels of key enzymes in THCA tumor samples. We randomly selected 382 patients from TCGA as the training dataset, and the remaining as the validation dataset. The 24 putative GSH metabolism related enzymes were included to construct the model via LASSO Cox regression algorithm. After 1,000-time alteration and cross-validation, a risk signature including *RRM1*, *GSR*, *GCLM*, *IDH2*, *RRM2*, *ODC1*, *GGT5*, *OPLAH* and *GSTZ1* was finally adopted. The exact parameters of the model were shown in **Figures 2A, B**. For this model, the Risk Score = 0.24×*GSTZ1* + 0.34×*GSR* + 0.09×*RRM1* + 0.65×*RRM2*-0.33×*IDH2*-0.11×*OPLAH*-0.52×*GCLM*-0.18×*GGT5*-0.25×*ODC1*. And we found that the risk score in patients who suffered from progression was significantly higher than that in those who did not in the training dataset (**Figure 2C**). The receiver operating characteristic (ROC) curve further confirmed the good prognostic prediction performance of our model (AUC = 0.888) (**Figure 2D**). Categorizing THCA patients into high and low groups based on the median of the risk score, we observed that patients with low-risk score achieved better DFS compared to those with high-risk score (P < 0.001, HR = 4.54, 95% CI:2.10-9.81) (**Figure 2E**). Univariate Cox proportional hazard regression analysis indicated the high-risk score was a negative prognostic factor of DFS for THCA (**Figure 2F**). We further applied the risk score model in the validation dataset to confirm its accuracy and stability. Similarly, high risk score was a negative prognostic factor for DFS (P < 0.001), and the AUC was 0.871 in predicting DFS status in ROC analysis (**Supplementary Figures S1A–D**).

Since many clinicopathological factors like age, TNM stage would also influence the survival outcomes of THCA, we conducted subgroup analysis in the entire TCGA cohort to further validate the robustness of the risk score model. Older patients, male patients and patients diagnosed with late stage tended to possess higher risk score (**Supplementary Figure S2**). High risk score remained as the negative prognostic factor for DFS in all subgroups (**Supplementary Figure S3**).

**Figure 3:**
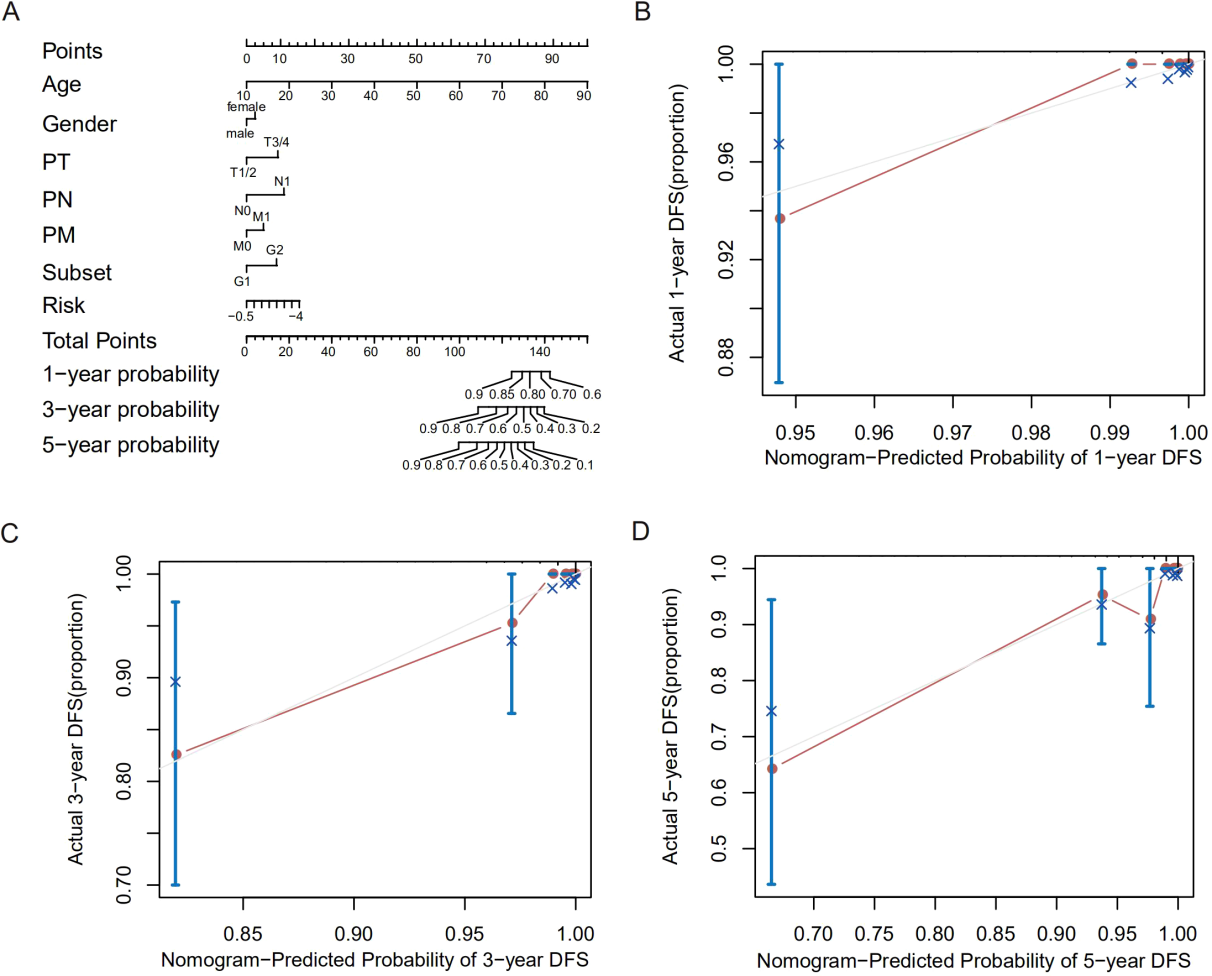
Construction and validation of a prognostic nomogram for DFS in THCA. **(A)** Nomogram integrating age, sex, and risk score to predict 1−, 3−, and 5−year DFS probabilities. **(B–D)** Calibration plots assessing the nomogram’s predictive accuracy for 1−year **(B)**, 3−year **(C)**, and 5−year **(D)** DFS probability.

We further constructed a nomogram to predict 1-, 3-, and 5-years DFS probability based on the risk score of the TCGA training dataset. Risk score deviated very little from actual DFS probability in nomogram analysis, such as 1-, 3- and 5-years DFS probability (**Figures 3A–D**). All these results supported that our risk score model could sensitively distinguish high-risk patients with dismal DFS from low-risk patients, thus might further instruct individualized treatment decisions.

### 3.3 Immune characteristics were distinct in the high- and low-risk patients

**Figure 4:**
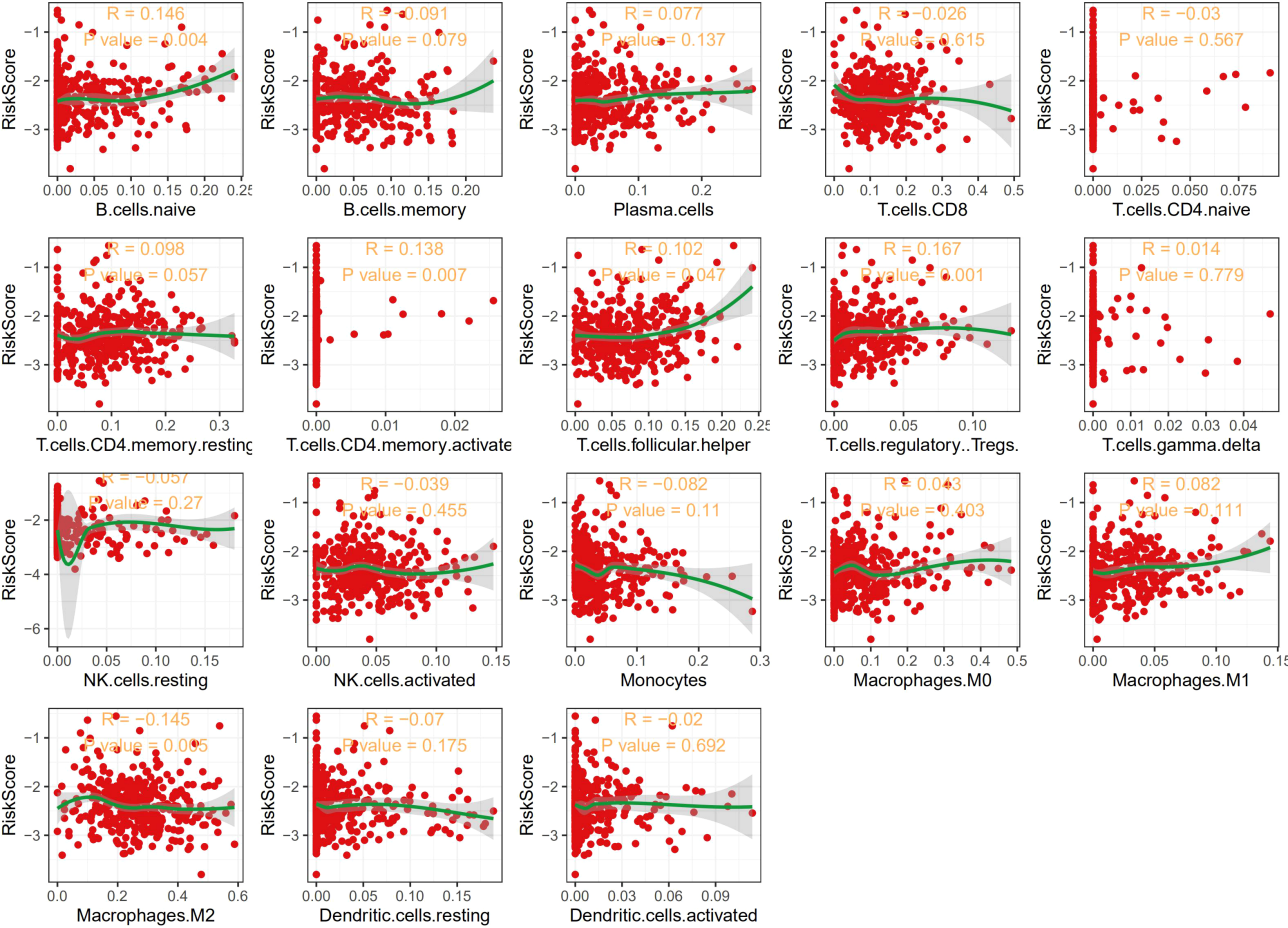
Correlation between different immune cell types and the risk score.

Previous research reported that GSH was essential for energy metabolism changes that were required for T cell effector functions (16). Thus, we explored the relationship between immune cells infiltration and the GSH metabolism related risk score. Decomposing 18 types of immune cells of THCA microenvironment using CIBERSORT, we observed that the risk score was positively correlated with proportion of naive B cells, activated memory CD4 T cells, follicular helper T cells and regulatory T cells (**Figure 4**).

### 3.4 High expression of RRM2 is associated with dismal survival outcomes and promotes proliferation and metastasis in THCA

By calculating the correlation between the risk scores of 510 thyroid cancer patients in the TCGA database and the RNA expression data of the nine genes included in the model, the results are shown in **Table 1**. The genes that have a high correlation with the risk scores and a significant *p*-value are the genes that best reflect the results of the prognostic model. From the table, we can see that the genes that are more suitable to be selected as experimental studies are, in order, *RRM2* (cor = 0.6310 p = 2.2627×10^-43^), ODC1 (cor = –0.4648 p = 1.1649×10^-21^), GCLM (cor = -0.4208 p = 1.1843×10^-17^), GGT5 (cor = -0.3876 p = 5.3263×10^-15^). Previous studies have shown that *RRM2* plays a crucial role in cell proliferation, invasion, migration, senescence, tumorigenesis, and other important cellular processes (17). High expression of *RRM2* has been associated with depressing survival outcomes in a variety of cancers, including breast cancer, gastric cancer, and bladder cancer. Since *RRM2* has also been found to be a risk factor for DFS in THCA, we further validated its effects on THCA tumor cells *in vitro* and *in vivo*.

**Table 1:** Correlation analysis of nine genes with risk score.

ID	Lasso-coef	Corr	Pvalue	Padj (Benjamini-Hochberg)	Padj (Bonferroni)
GSTZ1	0.2391	-0.0245	0.64	0.72	1
GSR	0.3379	-0.01	0.85	0.85	1
RRM1	0.0882	0.1442	4.96×10-3	6.38×10-3	0.045
RRM2	0.6533	0.631	2.26×10-43	2.03×10-42	2.03×10-42
IDH2	-0.3255	0.1846	3.07×10-4	5.53×10-4	2.76×10-3
OPLAH	-0.1085	-0.1473	4.11×10-3	6.17×10-3	0.037
GCLM	-0.5243	-0.4208	1.18×10-17	3.54×10-17	1.06×10-16
GGT5	-0.1825	-0.3876	5.33×10-15	1.20×10-14	4.80×10-14
ODC1	-0.2536	-0.4648	1.16×10-21	5.22×10-21	1.04×10-20

Correlations between the calculated risk score and mRNA expression levels of the nine model genes (n = 510) were assessed using Spearman’s rank correlation coefficient (two-sided). Resulting p-values were adjusted for multiple testing using both Benjamini–Hochberg false-discovery rate (FDR) correction and Bonferroni correction.

**Figure 5:**
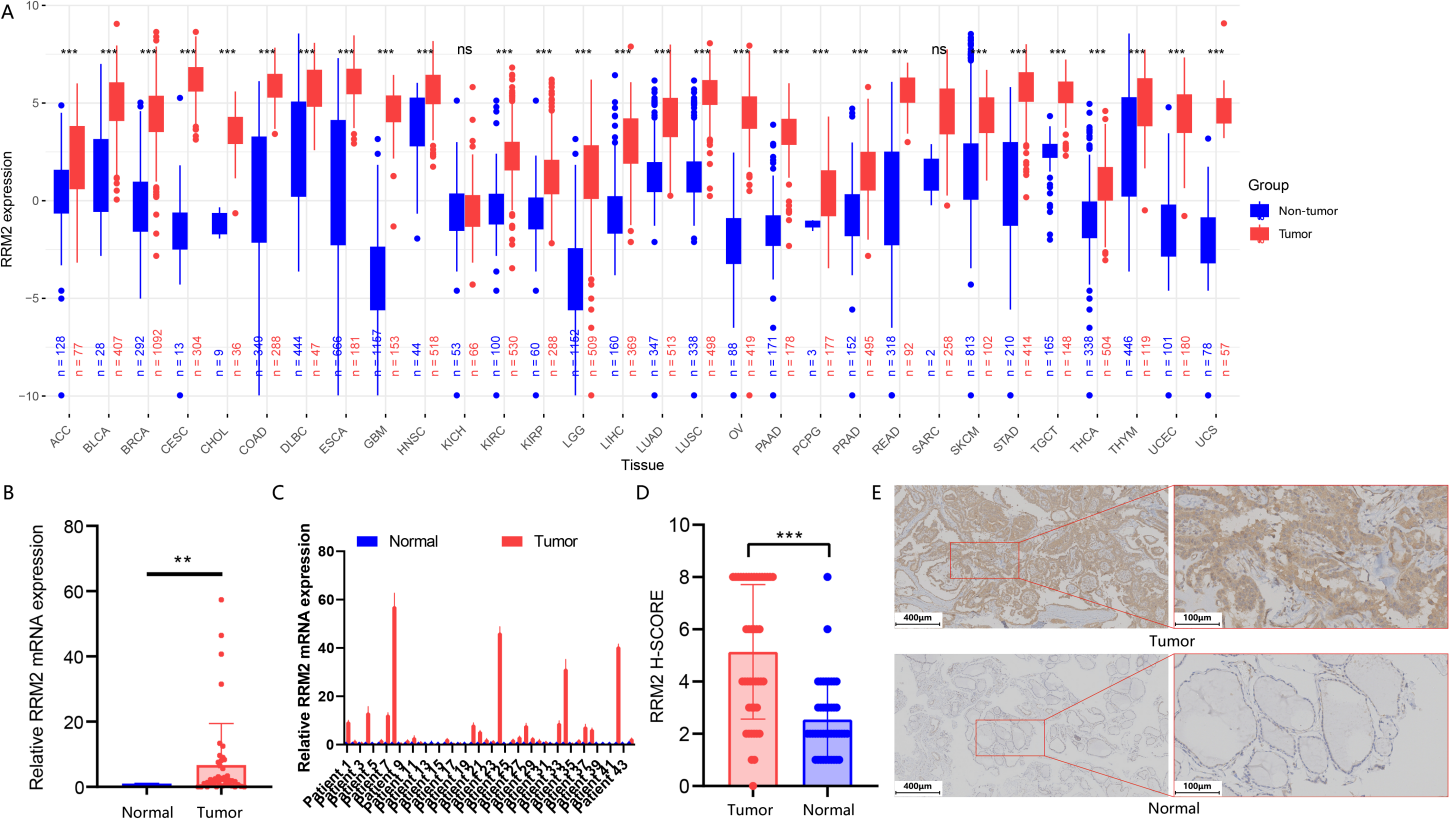
Pan−cancer and PTC–specific expression analysis of *RRM2*. **(A)** Differential expression of *RRM2* across 30 cancer types and their corresponding non-tumor tissues (tumor-adjacent tissues from the TCGA cohorts and non-diseased specimens from healthy donors in the GTEx database) based on TCGA and GTEx datasets, presented as log_2_ (TPM + 1); red, tumor; blue, non-tumor; ***p.adj < 0.001, ns (not significant). **(B)** Comparison of overall *RRM2* mRNA expression levels in PTC and paired adjacent thyroid epithelial tissues (**p < 0.01). **(C)** Relative *RRM2* mRNA expression in PTC tissues versus matched adjacent tissues. **(D)** Comparison of overall *RRM2* immunohistochemical expression in PTC and adjacent thyroid epithelial tissues (***p < 0.01). **(E)** Representative *RRM2* immunohistochemical staining in human PTC and adjacent tissues at 100×(scale bar, 200 µm) and 200×(scale bar, 100 µm) magnification.

In this study, we performed a systematic differential expression analysis of *RRM2* across 30 cancer types by integrating TCGA tumor data with both TCGA tumor-adjacent samples and non-diseased tissues from the GTEx database (**Figure 5A**). The results of the analysis were expressed as adjusted *p*-values (p.adj), and p.adj < 0.05 was considered statistically significant, full results are presented in **Supplementary Table 1**. The findings indicated that *RRM2* generally showed significantly high expression levels in most cancer types, especially in malignant tumors of epithelial origin, suggesting that it may play an important role in the development of these cancers. Notably, in THCA, *RRM2* also showed significant high expression (p.adj=4.90×10^43^), which may suggest a tumor-promoting role for *RRM2* in thyroid cancer.

We performed a detailed analysis of *RRM2* expression based on TNM stages and pathological types using the TCGA data, with the following key findings: Among the T stage, N stage, and M stage classifications, we found that *RRM2* expression was significantly correlated only with the N stage (**Supplementary Figures S4A–D**). Specifically, patients classified as N1 (indicating regional lymph node metastasis) showed higher *RRM2* expression than those classified as N0. When cases were grouped by pathological subtype, *RRM2* expression was higher in classic and tall-cell variants of papillary thyroid carcinoma compared with the follicular variant.

### 3.5 Expression levels of RRM2 in papillary thyroid carcinoma tissues and paired paracancerous thyroid tissues and their correlation with clinical features

The expression level of *RRM2* gene mRNA in each pair of tissues was detected by RT-qPCR. The results showed that the expression of *RRM2* was significantly higher in papillary thyroid carcinoma tissues than in paracancerous thyroid epithelial tissues (**Figure 5B**) (P<0.01), and this trend was verified in most samples (**Figure 5C**). Immunohistochemistry (IHC) was selected for the validation of *RRM2* protein expression in this patient cohort. The qPCR results were highly consistent with our subsequent IHC results. As shown in **Figure 5D**, the *RRM2* immunohistochemical score (H-SCORE) was significantly higher in PTC tissues than in paired paracancerous tissues (P<0.01), and the percentage of cancer tissues with higher *RRM2* H-SCORE than paracancerous tissues was 81.8% (36/44). Typical *RRM2* protein immunohistochemical results (**Figure 5E**) showed that in PTC tissues, deep staining of tan particles was observed, which were mainly aggregated in the cytoplasm. In contrast, paracarcinoma tissues were stained overall lighter and no obvious tan particles were seen. According to the American Joint Committee on Cancer/International Union Against Cancer (AJCC) TNM Staging System, Eighth Edition (TNM-8) criteria (18), the 2 ^-ΔΔ CT^>1 of the *RRM2* gene in the PTC tissues was used as the cut-off point for the expression level to classify the samples into the high-expression group and the low-expression group, and further analyses showed that, as shown in **Supplementary Table 2**, we found that the *RRM2* expression level was correlated with tumor size (P = 0.0048), which may indicate that *RRM2* acts as a potential oncogenic factor in PTC. Meanwhile, there was no significant correlation between its expression level and sex, age, TNM stage and tumor stage (P>0.05).

**Figure 6:**
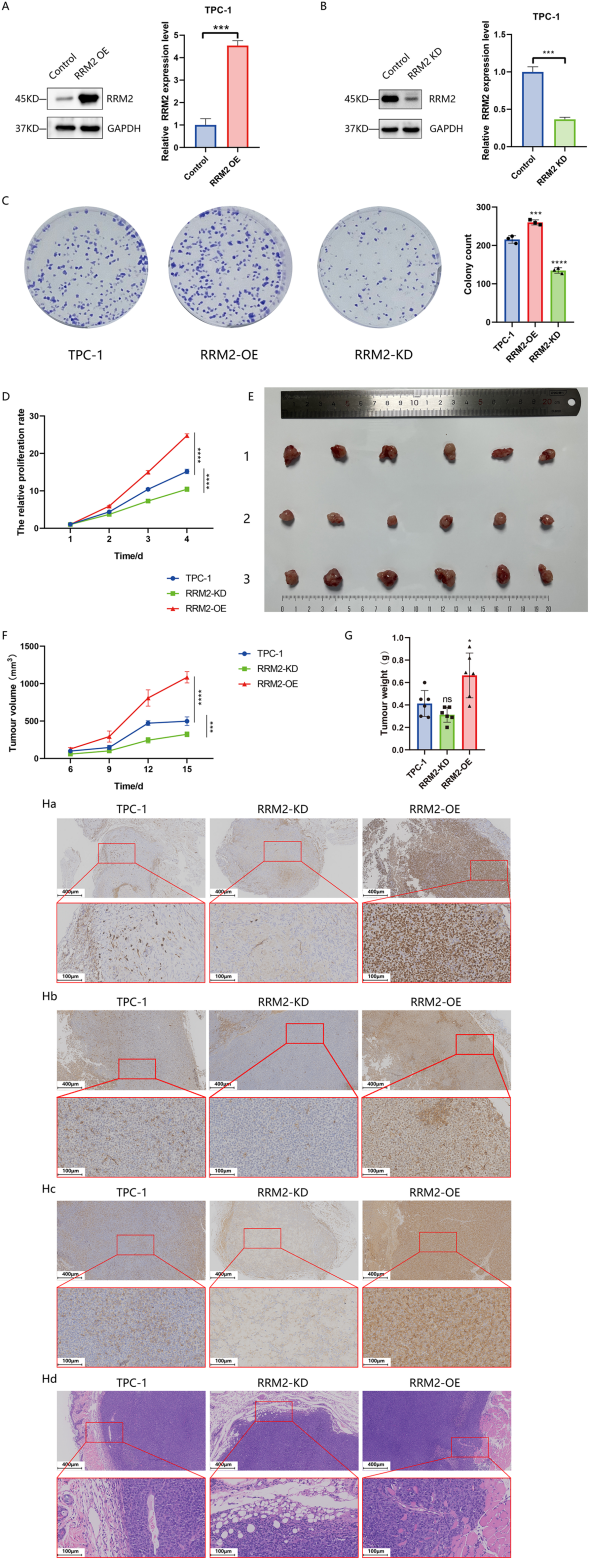
*RRM2* regulates PTC cell proliferation and tumor growth *in vitro* and *in vivo* (*p < 0.05, ***p < 0.001, ****p < 0.0001 and ns (not significant)). **(A, B)** Western blot analysis of *RRM2* protein levels in TPC−1 following *RRM2* knockdown (*RRM2*-KD) or overexpression (*RRM2*-OE), with GAPDH as loading control. **(C)** CCK−8 assay showing the effect of altered *RRM2* expression on TPC−1 cell proliferation over time (n = 3). **(D)** Effects of *RRM2* on TPC-1 cell colony formation detected by the plate colony formation assay(n=3). **(E)** Comparison of excised tumor volumes at endpoint between experimental and control groups (n = 6 per group), with tumor images shown from top to bottom (1): control (2), *RRM2*−KD (3), *RRM2*−OE. **(F)** Tumor growth curves i n nude mice over the experimental period (n = 6 per group), data presented as mean ± SEM; one−way ANOVA. **(G)** Comparison of final tumor weight between experimental and control groups(p < 0.05) (n=6 for each group) **(H)** Immunohistochemical and hematoxylin−eosin (HE) staining of tumors: upper panels at 100× (scale bar 200 µm) and lower panels at 200× (scale bar 100 µm). (Ha) Ki−67 staining: dense, uniform brown nuclei in *RRM2*−OE versus sparse staining in *RRM2*−KD tumors. (Hb) PCNA staining: strong, uniform signal in *RRM2*−OE versus near−absent in *RRM2*−KD. (Hc) *RRM2* staining: intense brown granules in *RRM2*−OE versus weak, non−specific staining in *RRM2*−KD. (Hd) HE staining confirming tumors’ histology with papillary structures, ground−glass nuclei, and adjacent skin, muscle, and adipose tissue.

Three *RRM2* knockdown cell lines (*RRM2*-KD-323, *RRM2*-KD-417, *RRM2*-KD-506) were successfully constructed using the lentiviral LV2N (U6/Puro) system and transfected with MOI = 15 in TPC-1 and IHH4 cells. RT-qPCR assays, as shown in **Supplementary Figures S4E, F**, compared with the wild-strain control, all three strains *RRM2* mRNA expression was significantly reduced in all *RRM2*-KD cells (P<0.01). Among them, the *RRM2*-KD-417 strain showed the highest knockdown efficiency and was therefore selected as a representative strain for subsequent experiments. The *RRM2* overexpression cell line (*RRM2*-OE) was successfully constructed by transfecting TPC-1 and IHH4 cells with MOI = 15 using lentiviral LV18 (CMV/Puro) system. RT-qPCR assay showed (**Supplementary Figures S4E, F**) that the expression level of *RRM2* mRNA was significantly higher in *RRM2*-OE cells than that in the wild-type control (P<0.01). The expression levels of *RRM2* protein in TPC-1 and *RRM2*-KD-TPC-1, TPC-1 and *RRM2*-OE-TPC-1, IHH4 and *RRM2*-KD-IHH4, IHH4 and *RRM2*-OE-IHH4 cells were detected by Western blot, respectively (**Figures 6A, B**; **Supplementary Figures S4G, H**).

CCK-8 assay and colony formation analysis showed that overexpression of *RRM2* promoted the proliferation of TPC-1 cell, whereas inhibition of *RRM2* expression reversed this process (**Figures 6C, D**). The subcutaneous loaded tumors in nude mice are shown in **Figure 6E**, **Supplementary Figure S5**, and the tumor growth curves (**Figure 6F**, **Supplementary Table 3**) showed that the experimental group *RRM2*-OE-TPC-1 had a steeper tumor growth curve and faster volume growth than the control group TPC-1, whereas the growth of tumors grown by the experimental group *RRM2*-KD-TPC-1 inoculation was significantly slowed down, with a downward shifting of the tumor volume growth curve (P<0.01). Finally, the average tumor volume of the *RRM2*-OE-TPC-1 group at the end of the experiment (1084.04 ± 74.91 mm³) was significantly larger than that of the control group (498.2 ± 57.84 mm³, P<0.01), while the average tumor volume of the *RRM2*-KD-TPC-1 group (322.93 ± 32.80 mm³) was significantly smaller than that of the control group (P<0.01). The results of tumor weighing (**Figure 6G**) showed that the tumor weight of the *RRM2*-OE-TPC-1 group was significantly higher than that of the control group (P<0.05), whereas there was no statistically significant difference between the *RRM2*-KD-TPC-1 group and the control group. By immunohistochemical analysis, Ki67 and PCNA expressions were significantly higher in the experimental *RRM2*-OE-TPC-1 group compared with the control group, while Ki67 and PCNA expressions were significantly lower in the experimental *RRM2*-KD-TPC-1 group (**Figure 6H**).

**Figure 7:**
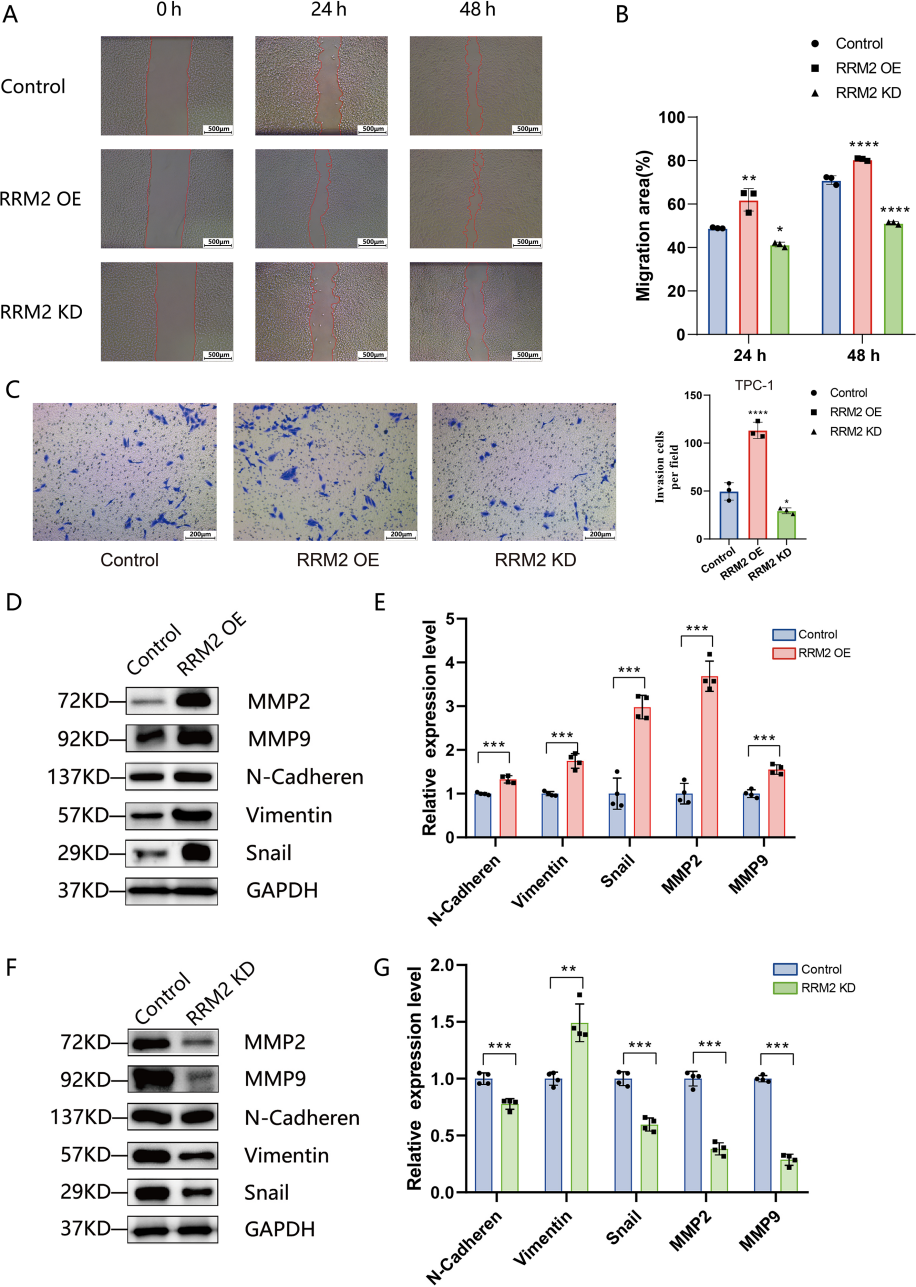
*RRM2* promotes migration, invasion, and EMT marker expression in PTC cells (*p < 0.05, **p < 0.01, ***p < 0.001 and ****p < 0.0001). **(A)** Wound−healing assay showing scratch closure in control TPC−1, *RRM2*−overexpressing (*RRM2*−OE), and *RRM2*−knockdown (*RRM2*−KD) cells at 0, 24, and 48 h. **(B)** Quantification of migration rates based on wound−closure area; data are mean ± SEM, one−way ANOVA. **(C)** Transwell invasion assay for control, *RRM2*−OE, *RRM2*−KD and TPC−1 cells; invaded cell counts are presented as mean ± SD (n = 3). **(D, E)** Western blot analysis of invasion−related proteins MMP2 and MMP9, and EMT markers N−Cadherin, Vimentin, and Snail in control versus *RRM2*−OE TPC−1 cells; GAPDH as loading control; data are mean ± SEM, Student’s t−test. **(F, G)** Western blot analysis of the same panel of MMP and EMT markers in control versus *RRM2*−KD TPC−1 cells; GAPDH loading control; data are mean ± SEM, Student’s t−test.

Given that *RRM2* could promote THCA proliferation *ex vivo*, we further explored whether *RRM2* could enhance the metastatic ability of THCA. Wound healing confirmed the enhanced migration of TPC-1 cells after *RRM2* overexpression. Twenty-four hours after scratching, *RRM2*-OE-TPC-1 cells migration by 13% (P<0.01), and 48 hours after scratching, *RRM2*-OE-TPC-1 cells in the experimental group increased migration by 8.86% (P<0.01), as shown in **Figures 7A, B**. In the Transwell invasion assay, a significant increase in the number of membrane-penetrating TPC-1 cells was observed in *RRM2* overexpressing group as compared to the control group, and a significant increase in the number of membrane-penetrating cells in the IHH4 cell line, a trend consistent with TPC-1 cells was observed (**Figure 7C**, **Supplementary Figure S6**). In contrast, knockdown of *RRM2* significantly inhibited the migration and invasion ability of THCA cells compared with the control group (**Figure 7C**, **Supplementary Figure S6**). The expression levels of matrix metalloproteinases (MMP2, MMP9) and markers related to epithelial mesenchymal transition (N-Cadherin, Vimentin, Snail) line were examined by Western blot. In TPC-1 cells, *RRM2* overexpression resulted in elevated protein expression levels of MMP2, MMP9, N-Cadherin, Vimentin, and Snail (P<0.01) (**Figures 7D, E**). In contrast, the expression levels of these proteins were significantly decreased after *RRM2* knockdown (P<0.01) (**Figures 7F, G**). Overall, *RRM2* promoted the growth of THCA cells and enhanced their metastatic ability, which is consistent with its negative prognostic effect on DFS in our dataset.

**Figure 8:**
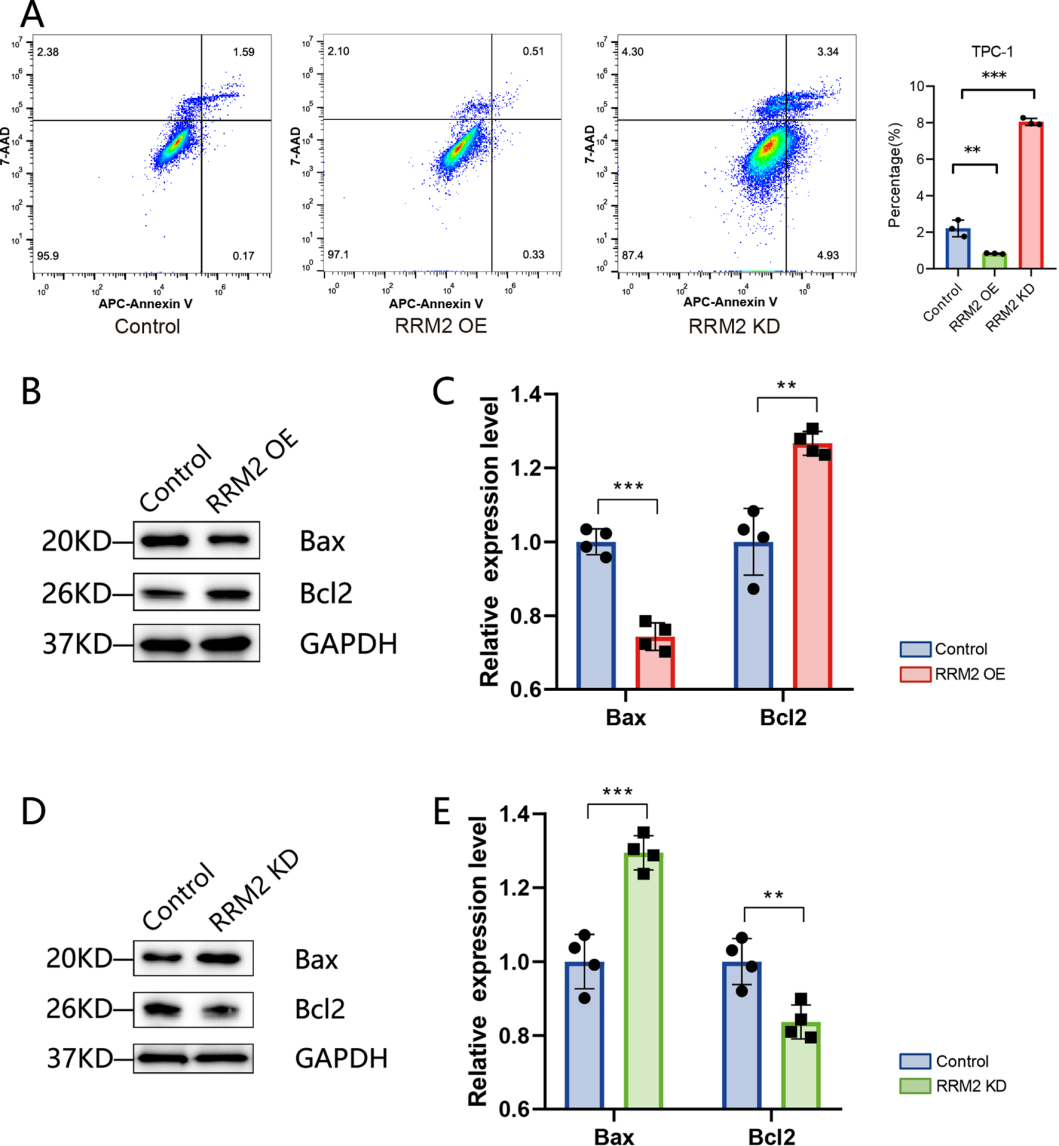
*RRM2* modulates apoptosis in TPC−1 cells (**p < 0.01 and ***p < 0.001). **(A)** Flow cytometric analysis of apoptosis in control, *RRM2*−overexpressing (*RRM2*−OE), and *RRM2*−knockdown (*RRM2*−KD) TPC−1 cells, showing distribution of early and late apoptotic populations. **(B, C)** Western blot of pro− and anti−apoptotic proteins Bax and Bcl−2 in control versus *RRM2*−OE TPC−1 cells; GAPDH as loading control; quantification presented as mean ± SEM, Student’s t−test. **(D, E)** Western blot of Bax and Bcl−2 in control versus *RRM2*−KD TPC−1 cells; GAPDH loading control; quantification presented as mean ± SEM, Student’s t−test.

In terms of apoptosis, the distribution of apoptosis in TPC-1 cells (control group), *RRM2*-KD-TPC-1 cells (knockdown group) and *RRM2*-OE-TPC-1 cells (overexpression group) was examined by flow cytometry (**Figure 8A**), and the results showed that apoptosis rate was reduced after overexpression of *RRM2* in the TPC-1 cell line compared with the control group (P< 0.01), and apoptosis rate was significantly increased after knockdown of *RRM2* (P<0.01). validation was consistent in IHH4 cell line (**Supplementary Figure S7**). In addition, we further validated the expression levels of apoptosis-related proteins, and the expression of Bax was significantly down-regulated (P<0.01), while the expression of Bcl-2 was significantly up-regulated (P<0.01) in *RRM2*-OE-TPC-1 cells compared to controls (**Figures 8B, C**). The results were opposite after knockdown of *RRM2* (**Figures 8D, E**).

**Figure 9:**
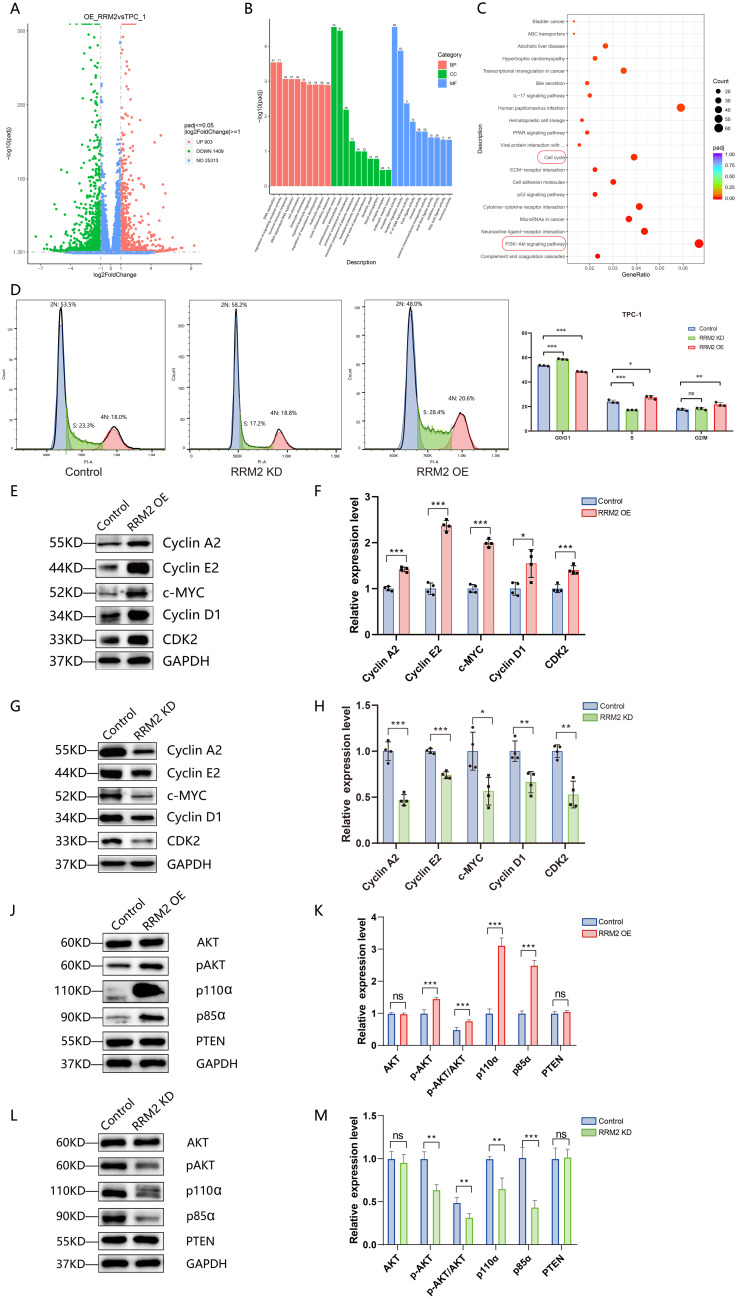
*RRM2* overexpression induces transcriptomic changes, influences cell cycle progression, and modulates Pl3K/Akt signaling in TPC-1 cells (*p < 0.05, **p < 0.01, ***p < 0.001 and ns (not significant)). **(A)** Volcano plot of differentially expressed genes (DEGs) in RRM2-OE versus control TPC-1 cells; red, significantly upregulated; blue, significantly downregulated (P < 0.05, |log_2_FC| > 1); gray, non-significant. **(B)** Gene Ontology (GO) enrichment analysis of DEGs, showing the top enriched biological processes, molecular functions, and cellular components; enrichment significance is plotted as –log_10_(adjusted P-value). **(C)** Kyoto Encyclopedia of Genes and Genomes (KEGG) pathway enrichment analysis of DEGs, highlighting tumor-related pathways such as PI3K-Akt and p53 signaling; enrichment significance is plotted as –log_10_(adjusted P-value). **(D)** Flow cytometric analysis of cell cycle distribution in control, *RRM2*−overexpressing (*RRM2*−OE), and *RRM2*−knockdown (*RRM2*−KD) TPC−1 cells; data are mean ± SD. **(E, F)** Western blot of cell cycle regulators Cyclin A2, Cyclin E2, c−MYC, Cyclin D1, and CDK2 in control versus *RRM2*−OE TPC−1 cells; GAPDH as loading control; quantification shown as mean ± SEM, Student’s t−test. **(G, H)** Western blot of the same panel of cell cycle proteins in control versus *RRM2*−KD TPC−1 cells; GAPDH loading control; data are mean ± SEM, Student’s t−test. **(J, K)** Western blot analysis of PI3K/Akt pathway proteins Akt, phosphorylated Akt (p−Akt), PI3K p85α, PI3K p110α, and PTEN in control versus *RRM2*−OE TPC−1 cells; GAPDH loading control; data are mean ± SEM, Student’s t−test. **(L, M)** Western blot analysis of the same PI3K/Akt signaling proteins in control versus *RRM2*−KD TPC−1 cells; GAPDH as loading control; data are mean ± SEM, Student’s t−test.

To investigate the effect of *RRM2* overexpression on the transcriptional level of papillary thyroid cancer cells, mRNA sequencing analysis was performed on wild strain TPC-1 cells and *RRM2* overexpression strain (*RRM2*-OE-TPC-1 cells) in this study. After normalizing the sequencing data, we screened a total of 2312 differentially expressed genes between control TPC-1 and experimental *RRM2*-OE-TPC-1 (**Figure 9A**). These differential genes were analyzed for GO and KEGG enrichment (**Figures 9B, C**), and GO was enriched in several biological processes, including DNA replication, regulation of signaling receptor activity, leukocyte chemotaxis and migration, etc. KEGG suggested that the PI3K-Akt signaling pathway and the cell cycle pathway were significantly enriched in the differential genes, and that *RRM2* might affect thyroid cancer cell proliferation and survival.

Analysis of mRNA sequencing results from wild strain TPC-1 cells and *RRM2* overexpression strain (*RRM2*-OE-TPC-1) showed significant enrichment of differential genes in the cell cycle pathway, as shown in **Supplementary Table 4**. To investigate the effect of *RRM2* on the cell cycle of THCA, we examined the effects of *RRM2* knockdown (*RRM2*-KD) and overexpression (*RRM2*-OE) on cell cycle distribution in the TPC-1 and IHH4 cell lines, respectively, using flow cytometry. As shown in **Figure 9D**, in TPC-1 cells, knockdown of *RRM2* resulted in a significantly higher proportion of cells in G0/G1 phase (P<0.01) and a significantly lower proportion of cells in S phase (P<0.01). In contrast, *RRM2* overexpression resulted in a significant decrease in the proportion of G0/G1 phase cells (P<0.01) and a significant increase in the proportion of S phase cells (P < 0.05). In IHH4 cells, we observed a similar trend (**Supplementary Figure S6**). These results suggest that *RRM2* may affect the cycle progression of thyroid cancer cells mainly by regulating the transition from G1 to S phase. To further elucidate the molecular mechanism of *RRM2* regulation of cell cycle, we examined the expression levels of cell cycle-related proteins (Cyclin A2, Cyclin E2, c-MYC, Cyclin D1, CDK2) in TPC-1 cells by Western blot. The expression levels of these proteins were significantly increased in the *RRM2* overexpression group (*RRM2*-OE-TPC-1) (P<0.05). The expression levels of these proteins in the *RRM2* overexpression group (*RRM2*-OE-TPC-1) were all significantly elevated (P < 0.05), with the most pronounced elevation of Cyclin E2 and c-MYC, as shown in **Figures 9E, F**. On the contrary, the protein expression levels of Cyclin A2, Cyclin E2, c-MYC, Cyclin D1, and CDK2 were all significantly reduced in the *RRM2* knockdown group (*RRM2*-KD-TPC-1) (P<0.05), with particularly significant decreases in Cyclin A2, c-MYC and CDK2, as shown in **Figures 9G, H**.

To explore the role of *RRM2* in the PI3K/Akt signaling pathway, we examined the expression levels of PI3K pathway-related proteins including Akt, phosphorylated Akt (pAkt), PI3K kinase p85α, PI3K kinase p110α, and PTEN in TPC-1, *RRM2* knockdown (*RRM2*-KD-TPC-1), and *RRM2* overexpression (*RRM2*-OE-TPC-1) cells by Western blot. The experimental results showed that *RRM2* overexpression or knockdown had no significant effect on the expression levels of Akt and PTEN proteins. However, the protein expression levels of pAkt, p85α, and p110α were significantly higher (P < 0.01) in the *RRM2* overexpression group (*RRM2*-OE-TPC-1) compared with the control group, as shown in **Figures 9J, K**. In contrast, the protein expression levels of pAkt, p85α, and p110α in the *RRM2* knockdown group (*RRM2*-KD-TPC-1) were significantly reduced (P < 0.01), see **Figures 9L, M**.

## 4 Discussions

Based on large THCA cohort from TCGA, we found that the GSH metabolism related enzymes were upregulated in tumor samples and negatively correlated with DFS. Thus, we for the first time built a risk stratification model based on the GSH metabolism related enzymes via LASSO Cox regression algorithm. Patients with high-risk score suffered from dismal DFS, exhibited a positive correlation with the infiltration levels of naïve B cells, activated memory CD4+ T cells, helper T cells, and regulatory T cells. The immune system responds to antigenic stimulation via dynamic shifts in lymphocyte subpopulations: B cells mediate humoral immunity, whereas CD4+ T cells serve as the “commanders” of the adaptive response. An increase in naïve B cells (or their recruitment) together with expansion of activated memory CD4+ T cells may reflect ongoing antigenic stimulation (for example, persistent tumor antigen exposure or chronic inflammation) and a degree of immune activation. The functional impact of increased helper T cells will depend on their polarization (Th1 versus Th2), which has divergent implications for antitumor immunity. Importantly, regulatory T cells (Tregs) play a vital immunosuppressive role in tumor immunity; by inhibiting effector T cell function and other antitumor immune mechanisms, Tregs facilitate immune evasion and tumor progression (19, 20). The enhanced Treg infiltration in this cohort indicates the development of tumor immunosuppression among thyroid cancer patients falling into the high-risk GSH-metabolism group. All these results indicated that the GSH metabolism related enzymes might play a pivotal role in THCA progression and was worth further researches.

Our risk model was developed and internally validated using TCGA mRNA expression data by randomly splitting the dataset into training and validation sets. We recognize that the current study lacks external validation using an independent RNA sequencing cohort. The primary reason for this limitation is the difficulty in identifying publicly available, high-quality thyroid cancer RNA sequencing datasets. These challenges stem from the need for datasets that are both technically homogenous (RNA-seq vs. microarray; differing normalization and batch effects) and possess sufficient corresponding clinical data (particularly long-term follow-up and survival status) to perform a reliable external validation of the prognostic risk score.

We fully understand that this absence of external validation limits the generalizability of our mRNA-based model. This limitation is expected to be addressed by a dedicated two-phased validation strategy in future work: Phase I Retrospective RNA Validation: This involves prioritizing an intensive search for and inclusion of any suitable independent external RNA-seq cohorts with adequate clinical follow-up data. A crucial step will be to perform a rigous retrospective validation after harmonized preprocessing (including consistent expression units and rigorous batch correction) to ensure the technical reliability of the analysis. Phase II Prospective Clinical Validation: A more robust, translational validation, focusing on the Asian population, is being prepared through a prospective, multi-center study. This phase will incorporate standardized sample processing and will include the evaluation of the nine model genes using Immunohistochemistry (IHC) scoring at the protein level, which is critical for direct clinical application. This phased approach is designed to systematically transition from computational mRNA-based findings to a clinically applicable protein-level diagnostic tool.

Based on the risk score model of glutathione metabolism-related enzyme genes in thyroid cancer, in a further correlation analysis, we screened the target gene *RRM2*, whose high expression was significantly and negatively correlated with disease-free survival of patients, from nine candidate genes. As one of the subunits of the nucleotide reductase enzyme, *RRM2* plays a key role in the process of DNA synthesis and repair. *RRM2* is aberrantly expressed in human tumors such as breast cancer (21, 22), gastric cancer (23), adrenocortical cancer (24), pancreatic cancer (25) and bladder cancer (26). Inhibition of *RRM2* can reduce RRase activity, inhibit cancer cell growth, promote cancer cell apoptosis, prevent tumor metastasis and reverse drug resistance. In breast cancer cells, it has been shown that *SNHG16*/miR-30a/*RRM2* and *TTN-AS1*/miR-524-5p/*RRM2* regulatory axes are involved in malignant proliferation and evasion of apoptosis in tumor cells, respectively (21, 22). In Huang’s study on non-small cell lung cancer (27), the results showed that AFAP1-AS1 up-regulated *RRM2* by inhibiting miR-139-5p expression, which in turn promoted the proliferation and chemotherapy tolerance of NSCLC cells. In addition, *RRM2* may exert its regulatory role by activating EGFR/AKT. In 2020, a related study (28) demonstrated that overexpression of miR-140-3p inhibited the proliferation of human cervical cancer cells by targeting down-regulation of *RRM2* inducing cell cycle arrest and early apoptosis, while decreasing *BCL-2* protein levels and inducing a significant increase in *BAX* and caspase-3 protein levels. In another THCA transcriptome data (29), it was also mentioned that it is closely associated as a core gene with the regulation of cell cycle and E2F-mediated DNA replication pathway. To further understand the role played by the *RRM2* gene in the whole biological behavior of tumors, we performed a pan-cancer analysis by the combination of TCGA and GTEx data, which showed that *RRM2* was generally highly expressed in tumors of epithelial origin, such as THCA, esophageal carcinoma, and breast carcinoma, suggesting that its pro-carcinogenic mechanism is conserved by the cancer species. We performed a detailed analysis of *RRM2* expression based on TNM stages and pathological types using the TCGA data, When cases were grouped by pathological subtype, *RRM2* expression was higher in classic and tall-cell variants of papillary thyroid carcinoma compared with the follicular variant. These observations are concordant with recent immunohistochemical data. For example, Ibrahim et al. reported negative *RRM2* staining in non-neoplastic thyroid tissue and non-invasive follicular thyroid neoplasm, whereas strong *RRM2* positivity was observed in PTC and invasive encapsulated follicular-variant PTC (30). Together, our findings and those of Ibrahim et al. support the hypothesis that *RRM2* is associated with more aggressive thyroid tumors and may therefore have potential utility as a biomarker to distinguish higher-risk or more invasive disease phenotypes. The correspondence between higher *RRM2* expression and N stage further reinforces this notion. The expression level of *RRM2* in PTC tissues was much higher than that in paracancerous thyroid epithelial tissues, and the expression level of *RRM2* was correlated with the size of the tumor (P<0.01). 44 cases of PTC tissues and paired paracancerous tissues diagnosed by pathology were selected, and the expression level of *RRM2* in PTC tissues was much higher than that in paracancerous thyroid epithelial tissues, and the expression level of *RRM2* was correlated with the size of the tumor (P<0.01). As mentioned above, *RRM2* plays an important role in the development of various cancers, but its effect on the biological behavior of THCA is still unclear and needs to be further explored.

*In vitro*, both CCK-8 assay and colony-formation assay showed that *RRM2* overexpression significantly increased the proliferation rate and clone formation ability of TPC-1 cells, while knockdown of *RRM2* significantly inhibited cell growth. Further *in vivo* nude mouse tumor formation experiments provided strong support for our *in vitro* observations. The experimental results showed that the tumor volume and weight of the *RRM2* overexpression group were significantly higher than those of the control group, and immunohistochemistry showed up-regulation of the expression of proliferation markers (e.g., Ki67, PCNA), which was in line with the previous observation that *RRM2* promotes cell proliferation in tumors such as breast and lung cancers, and further corroborated the promotional role of *RRM2* in tumorigenesis and development.

It has been pointed out in the literature (31, 32) that *RRM2* is involved in DNA synthesis and repair, and its high expression is often closely associated with cell cycle acceleration and high proliferation of tumor cells. In this study, we found by mRNA sequencing that a variety of genes involved in cell cycle regulation, DNA replication and mitosis were significantly up-regulated in *RRM2* overexpressing cells (*RRM2*-OE-TPC-1) in the experimental group, as shown in the **Supplementary Table 4**. This overall change in gene expression suggests that the intracellular network regulating cell cycle progression and DNA replication under *RRM2* overexpression conditions undergoes systematic remodeling, which may lead to abnormal acceleration of the cell cycle and sustained activation of proliferative signals.

Meanwhile, the results of Western blot assay showed that the protein expression levels of *Cyclin A2*, *Cyclin E2*, *c-MYC*, *Cyclin D1* and *CDK2* were significantly increased in the *RRM2* overexpression group, and *Cyclin D1*, *Cyclin E1/E2* and *Cyclin A2* are important regulators driving the cell cycle process, and their high expression is often associated with the acceleration of G1/S phase transition. *Cyclin D1* promotes the entry of cells from the G1 phase into the S phase in the early cell cycle (33), while *Cyclin E* and *Cyclin A* play key roles in the G1/S transition and S phase progression, respectively. The upregulation of these proteins in the Western blot data was highly consistent with the mRNA data, supporting the experimental group’s *RRM2*-OE-TPC-1 cells were in a state of continuous activation at various key nodes of the cell cycle, thus promoting rapid cell proliferation. In this regulatory network, the upregulation of *c-MYC*, as an important oncogenic transcription factor, not only directly promotes the expression of cell cycle proteins, but also participates in the regulation of cell metabolism, apoptosis, and proliferation, and other aspects of biological processes. *c-MYC* was elevated suggesting that *RRM2*-OE-TPC-1 cells may achieve the synergistic activation of multiple proliferative signaling pathways with the help of this transcription factor, accelerating tumor progression (34). In addition, the increased expression level of *CDK2*, which forms a complex with *Cyclin E* and *Cyclin A* and is a key kinase regulating the G1/S transition and S-phase progression, further corroborates the phenomenon of cell cycle deregulation.

Other genes detected in mRNA sequencing, such as *CDC20, CDC25A, BUB1, BUB1B*, and *MAD2L1*, are closely related to cell cycle checkpoints and fine regulation of chromosome segregation. Their abnormally high expression may predict increased chromosomal instability and abnormalities during cell division, thus providing a molecular basis for tumor aggressiveness and malignant transformation. Meanwhile, the upregulation of *MDM2* suggests that the p53 signaling pathway may be inhibited, which to a certain extent reduces the ability of cells to repair DNA damage and may prompt cells to evade apoptotic mechanisms, further accelerating tumor progression.

The results of Wound-healing assay and Transwell invasion assays showed that *RRM2* overexpression significantly enhanced the migration and invasion ability of TPC-1 cells, while knockdown of *RRM2* produced the opposite effect. Further Western blot analysis showed that *RRM2* regulated the expression levels of MMP2, MMP9 and EMT-related proteins (N-Cadherin, Vimentin, Snail), suggesting that *RRM2* may promote tumor cell invasion and metastasis by promoting matrix degradation and inducing epithelial mesenchymal transition.

TPC-1, *RRM2*-OE-TPC-1 mRNA sequencing and KEGG pathway analysis showed that the PI3K-Akt signaling pathway was significantly enriched in the differential genes. Western blot results further demonstrated that the expression level of *RRM2* was positively correlated with the levels of pAkt, PI3K p85α and p110α proteins. The total Akt expression level was unchanged, while the expression level of phosphorylated Akt (pAkt) was up-regulated with the overexpression of *RRM2*, suggesting that *RRM2* could be involved in the regulation of phosphorylation activation of Akt, which was consistent with the findings in other cancer types. The significant changes in PI3K kinases (subunits) p85α and p110α and the absence of significant changes in PTEN suggested that the *RRM2* regulation may activate Akt signaling through PI3K subunit stability regulation rather than the classical PTEN-dependent pathway.

In conclusion, we observed significant upregulation of GSH metabolism-related enzymes in THCA. For the first time, we established a risk stratification model based on GSH metabolism-related enzymes, and subsequently identified high-risk patient groups with poorer prognosis. Additionally, we identified *RRM2* as the key molecular candidate. In this study, we constructed a cell model of PTC with knockdown and overexpression of *RRM2*, and explored the specific functions of *RRM2* in the proliferation, migration, and invasion of PTC cells through a series of experiments. In the case of its impact on the tumor invasive biological behavioral phenotype, mRNA sequencing was applied to study the effect of its transcriptional level to further explore the downstream effector molecules or pathways of *RRM2*. Overall, the results suggest that *RRM2* acts as a pro-oncogenic molecule and may promote the progression of PTC by activating the PI3K/Akt signaling pathway, regulating key proteins of the cell cycle, and inducing the EMT process, which is in high agreement with previous findings in other malignant tumors. Nevertheless, the present study still has some limitations, as the current study has not yet externally validated our proposed risk stratification model, its clinical application still needs to be validated in external cohorts, including retrospective cohorts from other centers and future prospective multicenter cohorts. In addition, mRNA sequencing analysis suggested potential other signaling pathways such as p53 signaling pathway and IL -17 signaling pathway. In the future, we will combine Western blot and immunofluorescence experiments to purposefully elucidate the molecular mechanisms of PI3K, p53, and other candidate pathways in the progression of *RRM2*-mediated PTC, providing a more complete theoretical basis.

## Data Availability

The datasets presented in this study can be found in online repositories. The names of the repository/repositories and accession number(s) can be found below: https://www.ncbi.nlm.nih.gov/, GSE302548.
