# Supplementary material for: Developing a prognostic stratification model based on glutathione metabolism in thyroid cancer and validating RRM2’s tumor−promoting role
Source: Front Oncol. 2025 Nov 17;15:1700439. doi: 10.3389/fonc.2025.1700439 (PMC12665606; doi:10.3389/fonc.2025.1700439)
Supplement: Supplementary file 1 [file DataSheet1.docx]

Supplementary Material

# Supplementary Data

## Overexpression of *RRM2*

### Lentiviral construct composition and packaging system

Packaging plasmids and biosafety design: The lentiviral packaging system comprised three separate helper plasmids encoding the essential viral components: pGag/Pol (Gag and Pol), pRev (Rev) and pVSV-G (VSV-G envelope, replacing HIV-1 Env). These elements are provided on independent plasmids with no overlapping homologous sequences to minimize the risk of generating replication-competent lentivirus (RCL).

Recombinant transfer vector (LV18): The recombinant transfer plasmid LV18 contains a CMV (cytomegalovirus) promoter driving the transgene, a multiple cloning site (MCS) with unique restriction sites (e.g., BamHI, XhoI, NotI) downstream of the promoter for gene insertion, and a puromycin resistance cassette for selection. The full-length *RRM2* insert was cloned into the MCS under the CMV promoter; the resulting construct was verified by Sanger DNA sequencing prior to viral production.

### Experimental workflow for lentiviral production

Plasmid preparation: High-quality, endotoxin-free preparations of the transfer plasmid (LV18) and the three packaging plasmids were prepared using commercial kits and quantified prior to use.

Co-transfection and production: The transfer plasmid and the three helper plasmids were co-transfected into HEK293T cells using the transfection reagent RNAi-Mate (per manufacturer’s recommended protocol). Six hours after transfection, medium was replaced with complete growth medium to minimize cytotoxicity. Cells were incubated for 72 h to allow viral particle production.

Harvesting and concentration: At 72 h post-transfection, culture supernatants enriched for lentiviral particles were collected, clarified to remove cellular debris (e.g., low-speed centrifugation and filtration), and concentrated to obtain high-titer viral stocks. Concentrated viral aliquots were stored at −80 °C until use.

### Mechanistic notes on packaging and maturation

Rev binds RRE-containing RNA transcripts (including the transfer vector RNA and Gag/Pol mRNA) and mediates their nuclear export.

Gag/Pol proteins assemble in the cytoplasm to form the core structural scaffold, whereas VSV-G is incorporated into the producer cell membrane to pseudotype the viral particles.

Packaging specificity is driven by the Ψ (psi) packaging signal on the transfer vector RNA, ensuring selective encapsidation of the transfer RNA into budding particles.

Following budding, proteolytic processing of Gag/Pol yields mature, infectious lentiviral particles. (The corresponding procedure is illustrated in Supplementary Figure S7)

## sequences of all the shRNAs

Three independent shRNAs targeting human *RRM2* were used in this study:

*RRM2*-Homo-417: 5′-GAAACCCGAGGAGAGATATTT-3′

*RRM2*-Homo-506: 5′-TTAGCCAAGAAGTTCAGATTA-3′

*RRM2*-Homo-323: 5′-ATGATATCTGGCAGATGTATA-3′

A non-targeting negative control shRNA (shRNA-NC; scrambled control) was used with the sequence: 5′-TTCTCCGAACGTGTCACGT-3′.

# Supplementary Figures and Tables

## Supplementary Figures

**Figure S1. prognostic performance of the risk stratification model in the validation dataset.**

(A) Kaplan–Meier curve comparing DFS between high- and low-risk groups in the validation dataset.

(B) Box plot showing the distribution of risk scores between patients with disease progression and those without progression.

(C) ROC curve assessing the predictive accuracy of the risk stratification model, with an AUC of 0.871.

(D) Risk plot of THCA patients in the validation cohort. From top to bottom: distribution of risk scores in high- and low-risk groups, DFS status of each patient, and heatmap displaying the expression levels of nine GSH-related prognostic metabolic enzymes.

**Figure S2. risk score distribution across clinicopathological subgroups in the TCGA cohort.**

Box plots comparing the distribution of risk scores across subgroups stratified by clinical features, including age, sex, pathological stage, and TNM stage. Older patients, male patients, and those with advanced-stage disease exhibited significantly higher risk scores. Group comparisons of risk scores across clinical binary variables were performed primarily using one-way (single-factor) ANOVA given the large sample size (n = 510).

**Figure S3. prognostic value of the risk score model in different clinicopathological subgroups.**

Kaplan–Meier curves for DFS comparing high- and low-risk groups within various subgroups:

(A) Age subgroup;

(B) Sex subgroup;

(C) Pathological stage subgroup;

(D–F) TNM staging subgroups (T stage, N stage, M stage).

High risk score consistently predicted poorer DFS across all clinical subgroups, demonstrating the robustness of the prognostic model.

**Figure S4. clinicopathological Correlation of *RRM2* mRNA expression (TCGA Data) and Validation of *RRM2* Expression Alterations in TPC-1 and lHH4 Cell Lines.**

(A-D) Correlation of *RRM2* mRNA Expression with pathological types and TNM stages in Thyroid Cancer (TCGA Data).

(A) *RRM2* mRNA expression across different histological types of Thyroid Carcinoma: Classical PTC, Follicular PTC, Tall cell PTC, and other. Data were analyzed usingthe Kruskal-Wallis test (P= 0.005). The expression level is presented in FPKM. (* * P< 0.01; ns, not significant).(B) *RRM2* mRNA expression stratified by T stage (T1-T4). The overall difference was assessed bythe Kruskal-Wallis test (P= 0.735). (C) *RRM2* mRNA expression stratified by N stage (N0 vs. N1). A significant difference was observed using the Wilcoxon test (P= 0.008). (D) *RRM2* mRNA expression stratified by M stage (M0 vs. M1). The overall difference was assessed by the Wilcoxon test (P= 0.976).

(E-H) Validation of RRM2 knockdown and overexpression in TPC-1 and IHH4 cell lines.

(E–F) Relative mRNA expression levels of *RRM2* in three knockdown cell lines (*RRM2*-KD-323, *RRM2*-KD-417, and *RRM2*-KD-506) and in the overexpression cell line (*RRM2*-OE), as measured by RT–qPCR in TPC-1 and IHH4 cells. All knockdown cell lines exhibited significantly reduced *RRM2* expression compared to wild-type controls (P < 0.01), with *RRM2*-KD-417 demonstrating the highest knockdown efficiency. *RRM2*-OE cells showed significantly elevated *RRM2* mRNA levels compared to wild-type cells (P < 0.01).

(G–H) Western blot analysis of *RRM2* protein levels in IHH4 cells following *RRM2* knockdown (*RRM2*-KD) or overexpression (*RRM2*-OE). GAPDH was used as the loading control.

**Figure S5. tumor burden in nude mice (n = 6 per group)**

Tumor status of nude mice at the end of the experiment. Tumors from top to bottom correspond to: (1) TPC-1 control group, (2) *RRM2* knockdown group (*RRM2*-KD-TPC-1), and (3) *RRM2* overexpression group (*RRM2*-OE-TPC-1).

**Figure S6. impact of RRM2 expression alteration on the invasion, apoptosis, and cell cycle progression of IHH4 cells.**

1. Representative images and quantitative analysis of invaded cells in the control, RRM2 overexpression (RRM2-OE), RRM2 knockdown (RRM2-KD), and IHH4 groups. Data are presented as mean ± SD (n = 3). P < 0.01.

(B) Flow cytometric analysis of apoptosis in control, RRM2-overexpressing (RRM2-OE-IHH4), and RRM2-knockdown (RRM2-KD-IHH4) IHH4 cells. The distribution of early and late apoptotic cell populations was assessed to evaluate the impact of RRM2 expression on cell apoptosis.

(C) Flow cytometric analysis of cell cycle distribution in control, RRM2-overexpressing (RRM2-OE), and RRM2-knockdown (RRM2-KD) IHH4 cells. Data are presented as mean ± SD. P < 0.05, P < 0.01.

**Figure S7. the schematic diagram for lentiviral construct, packaging system and production workflow used for *RRM2* overexpression**

(A–D) Circular maps of the recombinant transfer vector and the three helper (packaging) plasmids.

1. Experimental workflow for lentiviral production.
2. Mechanistic overview of lentiviral packaging and maturation.

## Supplementary Tables

**Supplementary table I. differential expression analysis of RRM2 across pan-cancer tissues**

| **Tissue** | **group1** | **group2** | **p** | **p.adj** |
| --- | --- | --- | --- | --- |
| Adrenocortical Carcinoma (ACC) | normal | tumor | 1.71×10-7 | 1.00×10-6 |
| Bladder Urothelial Carcinoma (BLCA) | normal | tumor | 2.12×10-8 | 1.50×10-7 |
| Breast Carcinoma (BRCA) | normal | tumor | 1.34×10-144 | 3.50×10-143 |
| Cervical Squamous Cell Carcinoma and Endocervical Adenocarcinoma (CESC) | normal | tumor | 3.55×10-6 | 1.40×10-5 |
| Cholangiocarcinoma (CHOL) | normal | tumor | 1.67×10-14 | 1.50×10-13 |
| Colon Adenocarcinoma (COAD) | normal | tumor | 5.94×10^-^100 | 1.40×10-98 |
| Diffuse Large B-Cell Lymphoma (DLBC) | normal | tumor | 2.02×10-24 | 2.00×10-23 |
| Esophageal Carcinoma (ESCA) | normal | tumor | 1.99×10^-^161 | 5.40×10-160 |
| Glioblastoma Multiforme (GBM) | normal | tumor | 7.92×10^-^249 | 2.30×10-247 |
| Head and Neck Squamous Cell Carcinoma (HNSC) | normal | tumor | 2.46×10-7 | 1.20×10-6 |
| Kidney Chromophobe (KICH) | normal | tumor | 0.37 | 0.58 |
| Kidney Renal Clear Cell Carcinoma (KIRC) | normal | tumor | 5.62×10-27 | 6.70×10-26 |
| Kidney Renal Papillary Cell Carcinoma (KIRP) | normal | tumor | 9.28×10-11 | 7.40×10-10 |
| Brain Lower Grade Glioma (LGG) | normal | tumor | 6.17×10-250 | 1.80×10-248 |
| Liver Hepatocellular Carcinoma (LIHC) | normal | tumor | 6.27×10-57 | 1.20×10-55 |
| Lung Adenocarcinoma (LUAD) | normal | tumor | 1.22×10^-^120 | 3.10×10-119 |
| Lung Squamous Cell Carcinoma (LUSC) | normal | tumor | 2.02×10^-^177 | 5.70×10-176 |
| Ovarian carcinoma (OV) | normal | tumor | 6.86×10-44 | 1.00×10-42 |
| Pancreatic Adenocarcinoma (PAAD) | normal | tumor | 5.11×10-94 | 1.20×10-92 |
| Pheochromocytoma and Paraganglioma (PCPG) | normal | tumor | 9.68×10-4 | 2.90×10-3 |
| Prostate Adenocarcinoma (PRAD) | normal | tumor | 2.23×10-32 | 2.90×10-31 |
| Rectum Adenocarcinoma (READ) | normal | tumor | 1.62×10-92 | 3.60×10-91 |
| Sarcoma (SARC) | normal | tumor | 0.29 | 0.58 |
| Skin Cutaneous Melanoma (SKCM) | normal | tumor | 1.89×10-41 | 2.60×10-40 |
| Stomach Adenocarcinoma (STAD) | normal | tumor | 1.62×10-67 | 3.20×10-66 |
| Testicular Germ Cell Tumors (TGCT) | normal | tumor | 2.30×10-88 | 4.80×10-87 |
| Thyroid Carcinoma (THCA) | normal | tumor | 3.09×10-44 | 4.90×10-43 |
| Thymoma (THYM) | normal | tumor | 1.21×10-24 | 1.30×10-23 |
| Uterine Corpus Endometrial Carcinoma (UCEC) | normal | tumor | 1.12×10-53 | 2.00×10-52 |
| Uterine Carcinosarcoma (UCS) | normal | tumor | 4.20×10-50 | 7.10×10-49 |

Gene expression differences between tumor and normal tissues were assessed for each tissue type using two-sided Welch’s t-tests. P-values were adjusted for multiple testing across tissue types using the Holm method.

**Supplementary table II. association between *RRM2* expression levels and clinical characteristics in PTC tissues (n = 44)**

| **Clinical Characteristics** | **High RRM2 Expression (n = 29)** | **Low RRM2 Expression (n = 15)** | **P-value** |
| --- | --- | --- | --- |
|  |  |  |  |
| Sex |  |  | 0.9999 |
| Male | 12 | 6 |  |
| Female | 17 | 9 |  |
| Age |  |  | 0.7222 |
| < 55 years | 21 | 12 |  |
| ≥ 55 years | 8 | 3 |  |
| Tumor Size |  |  | 0.0048 |
| ＜2cm | 12 | 13 |  |
| ≥2cm | 17 | 2 |  |
| T Stage |  |  | 0.067 |
| T1–T2 | 19 | 14 |  |
| T3–T4 | 10 | 1 |  |
| Lymph Node Metastasis (N Stage) |  |  | 0.3944 |
| Negative | 3 | 3 |  |
| Positive | 26 | 12 |  |
| TNM Stage |  |  | 0.2308 |
| Stage I–II | 22 | 14 |  |
| Stage III–IV | 7 | 1 |  |

Categorical variables were compared using Fisher’s exact test (two-sided).

**Supplementary table III. tumor measurements**

| **Mouse ID** | **Group** | **Days Post Inoculation(Day)** | **Long Diameter (mm)** | **Short Diameter (mm)** | **Volume (L²×W/2, mm^3^)** |
| --- | --- | --- | --- | --- | --- |
| C31 | RRM2-KD | 10 | 5.09 | 5.08 | 65.807 |
| C31 | RRM2-KD | 13 | 6.6 | 5.18 | 112.82 |
| C31 | RRM2-KD | 16 | 8.58 | 6.75 | 248.455 |
| C31 | RRM2-KD | 19 | 9.4 | 8.2 | 362.276 |
| C32 | RRM2-KD | 10 | 4.59 | 4.22 | 44.454 |
| C32 | RRM2-KD | 13 | 6.5 | 5.56 | 117.455 |
| C32 | RRM2-KD | 16 | 8.78 | 7.1 | 273.664 |
| C32 | RRM2-KD | 19 | 9.5 | 7.6 | 342.95 |
| C33 | RRM2-KD | 10 | 5 | 4.4 | 55 |
| C33 | RRM2-KD | 13 | 5.97 | 4.53 | 80.727 |
| C33 | RRM2-KD | 16 | 8.13 | 8.1 | 267.692 |
| C33 | RRM2-KD | 19 | 8.5 | 7.6 | 274.55 |
| C34 | RRM2-KD | 10 | 5.17 | 4.09 | 54.661 |
| C34 | RRM2-KD | 13 | 6.74 | 4.89 | 111.07 |
| C34 | RRM2-KD | 16 | 7.93 | 6.57 | 206.577 |
| C34 | RRM2-KD | 19 | 9.2 | 8.1 | 342.792 |
| C35 | RRM2-KD | 10 | 5.06 | 4.6 | 58.888 |
| C35 | RRM2-KD | 13 | 6.06 | 5.3 | 97.318 |
| C35 | RRM2-KD | 16 | 7.43 | 6.89 | 190.181 |
| C35 | RRM2-KD | 19 | 8.9 | 7.5 | 297.038 |
| C36 | RRM2-KD | 10 | 5.59 | 4.88 | 76.245 |
| C36 | RRM2-KD | 13 | 5.81 | 5.62 | 94.855 |
| C36 | RRM2-KD | 16 | 8.72 | 7.29 | 277.16 |
| C36 | RRM2-KD | 19 | 9.6 | 6.9 | 317.952 |
| C41 | RRM2-OE | 10 | 6.53 | 5.95 | 126.857 |
| C41 | RRM2-OE | 13 | 8.99 | 8.66 | 349.951 |
| C41 | RRM2-OE | 16 | 15.15 | 7.21 | 827.429 |
| C41 | RRM2-OE | 19 | 15.2 | 9.9 | 1143.648 |
| C42 | RRM2-OE | 10 | 6.7 | 5.04 | 113.123 |
| C42 | RRM2-OE | 13 | 9.19 | 7.55 | 318.822 |
| C42 | RRM2-OE | 16 | 14.86 | 7.61 | 840.219 |
| C42 | RRM2-OE | 19 | 14.1 | 11.4 | 1133.217 |
| C43 | RRM2-OE | 10 | 6.46 | 5.52 | 115.179 |
| C43 | RRM2-OE | 13 | 9.77 | 8.34 | 398.039 |
| C43 | RRM2-OE | 16 | 15.22 | 6.64 | 769.073 |
| C43 | RRM2-OE | 19 | 15.1 | 9.2 | 1048.846 |
| C44 | RRM2-OE | 10 | 6.28 | 5.82 | 114.766 |
| C44 | RRM2-OE | 13 | 8.09 | 7.05 | 230.705 |
| C44 | RRM2-OE | 16 | 12.59 | 12.06 | 955.804 |
| C44 | RRM2-OE | 19 | 14.8 | 10.7 | 1171.864 |
| C45 | RRM2-OE | 10 | 6.78 | 5.68 | 130.55 |
| C45 | RRM2-OE | 13 | 8.14 | 7.36 | 243.835 |
| C45 | RRM2-OE | 16 | 11.98 | 8.7 | 624.314 |
| C45 | RRM2-OE | 19 | 15.4 | 8.4 | 996.072 |
| C46 | RRM2-OE | 13 | 8.27 | 6.38 | 218.173 |
| C46 | RRM2-OE | 16 | 14.13 | 8.34 | 832.569 |
| C46 | RRM2-OE | 19 | 13.2 | 11.6 | 1010.592 |
| C21 | TPC-1 | 10 | 6.29 | 4.49 | 88.821 |
| C21 | TPC-1 | 13 | 7.24 | 6.12 | 160.398 |
| C21 | TPC-1 | 16 | 10.13 | 8.26 | 423.808 |
| C21 | TPC-1 | 19 | 10.7 | 8.4 | 480.858 |
| C22 | TPC-1 | 10 | 5.77 | 5.56 | 92.554 |
| C22 | TPC-1 | 13 | 7.38 | 5.88 | 160.125 |
| C22 | TPC-1 | 16 | 10.55 | 8.34 | 464.131 |
| C22 | TPC-1 | 19 | 11.5 | 8.7 | 575.288 |
| C23 | TPC-1 | 10 | 6.34 | 5.16 | 103.705 |
| C23 | TPC-1 | 13 | 7.54 | 6.51 | 185.052 |
| C23 | TPC-1 | 16 | 10.19 | 9.16 | 475.569 |
| C23 | TPC-1 | 19 | 9.7 | 9.5 | 446.928 |
| C24 | TPC-1 | 10 | 5.72 | 4.41 | 72.144 |
| C24 | TPC-1 | 13 | 7.14 | 5.59 | 142.488 |
| C24 | TPC-1 | 16 | 10.29 | 8.94 | 473.302 |
| C24 | TPC-1 | 19 | 10.8 | 9.7 | 565.704 |
| C25 | TPC-1 | 10 | 6.43 | 6.05 | 125.068 |
| C25 | TPC-1 | 13 | 6.53 | 6.13 | 130.694 |
| C25 | TPC-1 | 16 | 11.11 | 7.68 | 473.979 |
| C25 | TPC-1 | 19 | 10.3 | 8.4 | 445.578 |
| C26 | TPC-1 | 10 | 6.53 | 5.48 | 116.836 |
| C26 | TPC-1 | 13 | 5.93 | 5.88 | 103.385 |
| C26 | TPC-1 | 16 | 10.46 | 9.57 | 523.535 |
| C26 | TPC-1 | 19 | 10.7 | 8.3 | 475.134 |

**Supplementary table IV. differential expression analysis of cell cycle–related genes between *RRM2*-overexpressing and wild-type TPC-1 cells**

| **ID** | **RRM2-OE** | **TPC-1** | **log2FoldChange** | **padj** | **gene_description** |
| --- | --- | --- | --- | --- | --- |
| CCND1 | 14176.1356 | 5774.2346 | 1.2958 | 0 | cyclin D1 [Source: HGNC Symbol; Acc: HGNC: 1582] |
| CDKN1A | 10144.2614 | 2011.4887 | 2.3344 | 0 | cyclin dependent kinase inhibitor 1A [Source: HGNC Symbol; Acc: HGNC: 1784] |
| MCM4 | 7312.8282 | 2611.1624 | 1.4854 | 0 | minichromosome maintenance complex component 4 [Source: HGNC Symbol; Acc: HGNC: 6947] |
| MDM2 | 6439.5263 | 1413.7265 | 2.1872 | 0 | MDM2 proto-oncogene [Source: HGNC Symbol; Acc: HGNC: 6973] |
| PCNA | 2867.9616 | 804.3011 | 1.8337 | 2.53×10-248 | proliferating cell nuclear antigen [Source: HGNC Symbol; Acc: HGNC:8729] |
| CDC20 | 3778.7212 | 1486.3673 | 1.3462 | 1.72×10-160 | cell division cycle 20 [Source: HGNC Symbol; Acc: HGNC: 1723] |
| YWHAH | 3158.0299 | 1498.7027 | 1.0751 | 3.24×10-118 | tyrosine 3-monooxygenase /tryptophan 5-monooxygenase activation protein eta [Source: HGNC Symbol; Acc: HGNC: 12853] |
| CDK6 | 2468.5351 | 1039.5906 | 1.2476 | 1.68×10-91 | cyclin dependent kinase 6 [Source: HGNC Symbol; Acc: HGNC: 1777] |
| CDC6 | 1200.9866 | 455.3838 | 1.3997 | 8.85×10-77 | cell division cycle 6 [Source: HGNC Symbol; Acc:HGNC: 1744] |
| TRIP13 | 1161.4374 | 384.9845 | 1.5918 | 3.90×10-75 | thyroid hormone receptor interactor 13 [Source: HGNC Symbol; Acc: HGNC: 12307] |
| CDCA5 | 1225.3236 | 467.5061 | 1.3902 | 3.27×10-74 | cell division cycle associated 5 [Source: HGNC Symbol; Acc: HGNC: 14626] |
| BUB1 | 2006.7767 | 985.3603 | 1.0264 | 7.75×10-73 | BUB1 mitotic checkpoint serine/ threonine kinase [Source: HGNC Symbol; Acc: HGNC: 1148] |
| SKP2 | 964.9282 | 339.2931 | 1.5082 | 5.57×10-71 | S-phase kinase associated protein 2 [Source: HGNC Symbol; Acc: HGNC: 10901] |
| CCNA2 | 1593.5587 | 685.8466 | 1.2162 | 1.65×10-67 | cyclin A2 [Source: HGNC Symbol; Acc: HGNC: 1578] |
| KNL1 | 1413.7583 | 636.4083 | 1.1518 | 1.66×10-60 | kinetochore scaffold 1 [Source: HGNC Symbol; Acc: HGNC: 24054] |
| MAD2L1 | 1549.5971 | 739.3431 | 1.068 | 5.29×10-57 | mitotic arrest deficient 2 like 1 [Source: HGNC Symbol; Acc: HGNC: 6763] |
| CDC25A | 667.5561 | 218.0737 | 1.6148 | 1.94×10-54 | cell division cycle 25A [Source: HGNC Symbol; Acc: HGNC: 1725] |
| PPP2R1B | 1315.8884 | 643.1599 | 1.0329 | 2.04×10-52 | protein phosphatase 2 scaffold subunit Abeta [Source: HGNC Symbol; Acc: HGNC: 9303] |
| BUB1B | 1414.2098 | 680.6644 | 1.0548 | 5.55×10-47 | BUB1 mitotic checkpoint serine/ threonine kinase B [Source: HGNC Symbol; Acc: HGNC: 1149] |
| ORC1 | 494.7778 | 129.8351 | 1.9313 | 7.41×10-47 | origin recognition complex subunit 1 [Source: HGNC Symbol; Acc: HGNC: 8487] |
| CCNE2 | 399.474 | 104.23 | 1.9384 | 3.01×10-42 | cyclin E2 [Source: HGNC Symbol; Acc: HGNC: 1590] |
| PTTG1 | 890.3242 | 414.1176 | 1.1037 | 3.92×10-36 | pituitary tumor-transforming 1 [Source: HGNC Symbol; Acc: HGNC: 9690] |
| TTK | 863.8772 | 409.0504 | 1.0799 | 1.56×10-35 | TTK protein kinase [Source: HGNC Symbol; Acc: HGNC: 12401] |
| ORC6 | 709.684 | 303.479 | 1.2243 | 3.93×10-35 | origin recognition complex subunit 6 [Source: HGNC Symbol; Acc: HGNC: 17151] |
| ORC3 | 638.5865 | 254.5921 | 1.3274 | 4.64×10-35 | origin recognition complex subunit 3 [Source: HGNC Symbol; Acc: HGNC: 8489] |
| TICRR | 621.1276 | 282.0657 | 1.1391 | 6.31×10-29 | TOPBP1 interacting checkpoint and replication regulator [Source: HGNC Symbol; Acc: HGNC: 28704] |
| CDC45 | 520.5013 | 251.7822 | 1.0478 | 5.46×10-22 | cell division cycle 45 [Source: HGNC Symbol; Acc: HGNC: 1739] |
| CCNE1 | 137.9562 | 47.9688 | 1.5231 | 2.15×10-11 | cyclin E1 [Source: HGNC Symbol; Acc: HGNC: 1589] |
| SFN | 14.5942 | 1.5944 | 3.1832 | 1.63×10-3 | stratifin [Source: HGNC Symbol; Acc: HGNC: 10773] |
| CCNA1 | 21.7804 | 47.5664 | -1.1229 | 3.43×10-3 | cyclin A1 [Source: HGNC Symbol; Acc: HGNC: 1577] |
