# Supplementary figures and images for: Developing a prognostic stratification model based on glutathione metabolism in thyroid cancer and validating RRM2’s tumor−promoting role
Source: Front Oncol. 2025 Nov 17;15:1700439. doi: 10.3389/fonc.2025.1700439 (PMC12665606; doi:10.3389/fonc.2025.1700439)

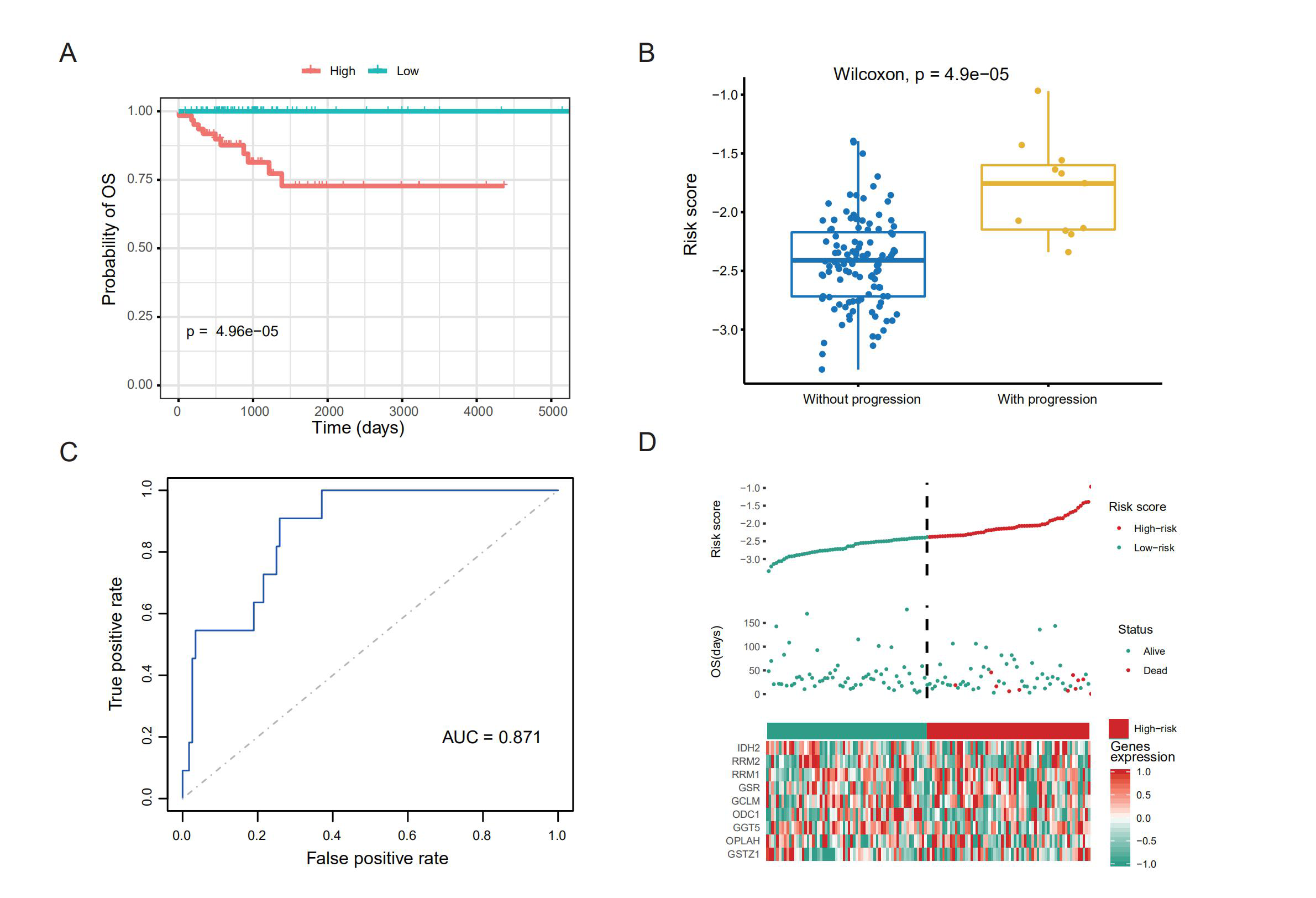

Supplement: Supplementary file 2 [file Image1.tif]

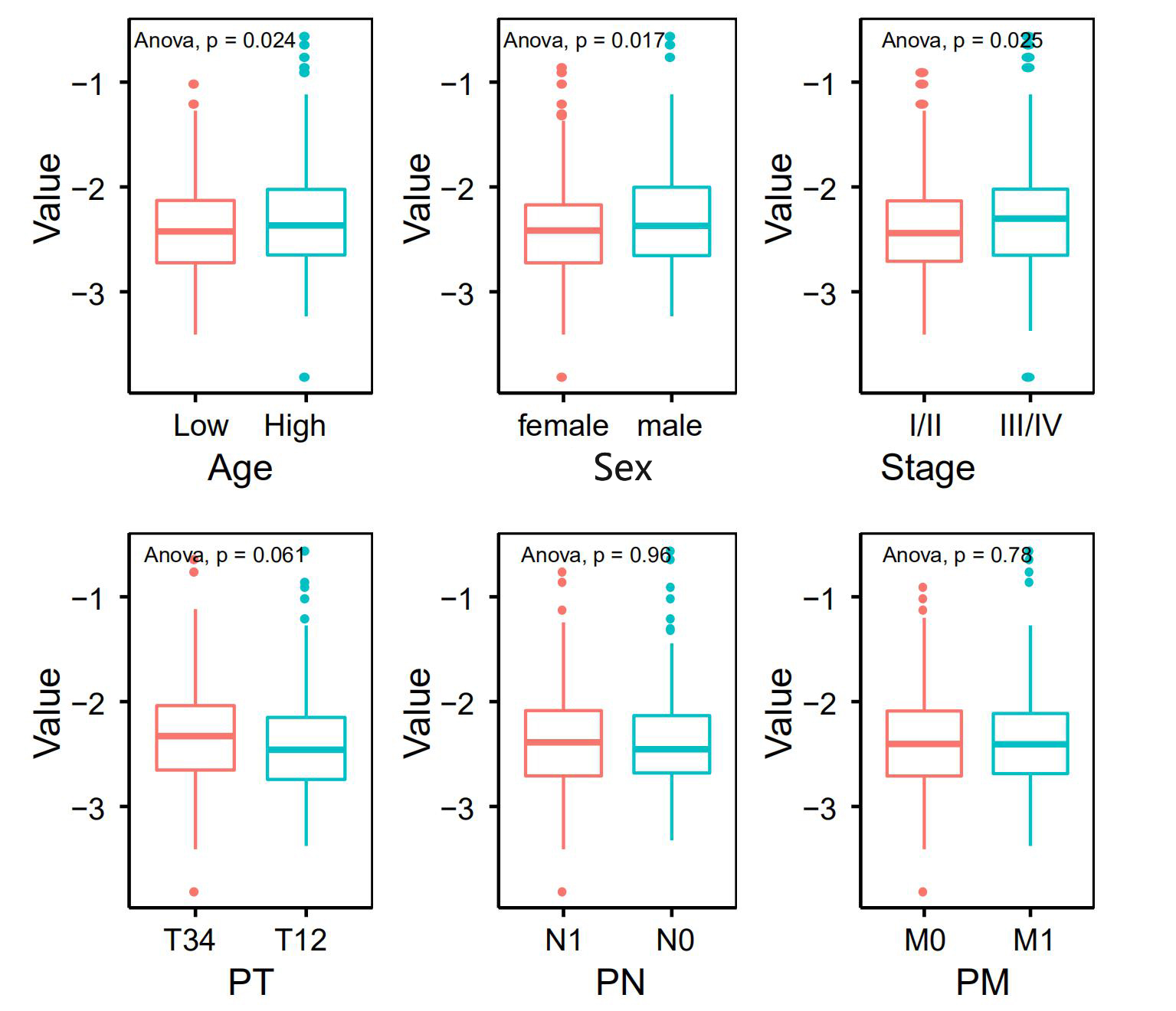

Supplement: Supplementary file 3 [file Image2.tif]

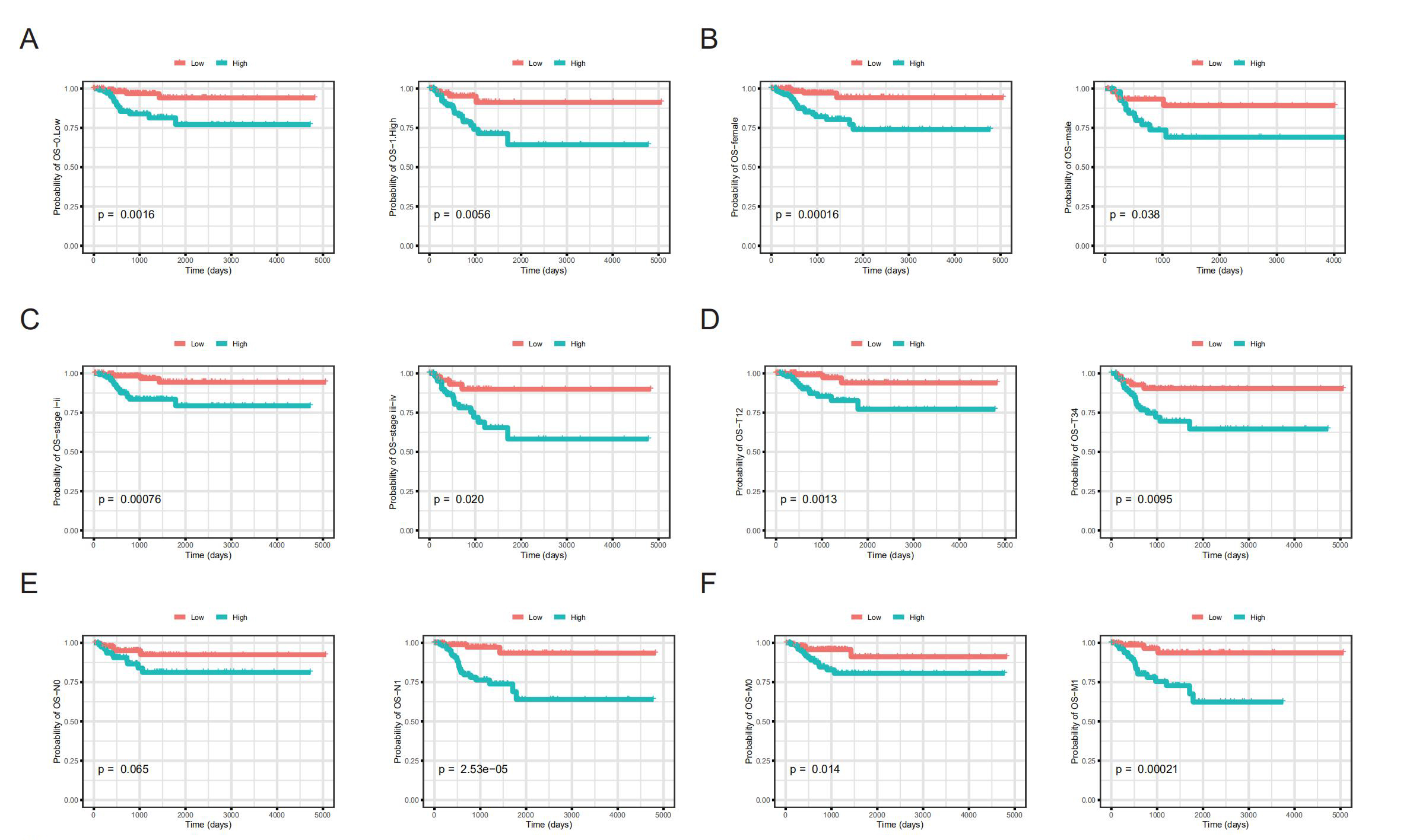

Supplement: Supplementary file 4 [file Image3.tif]

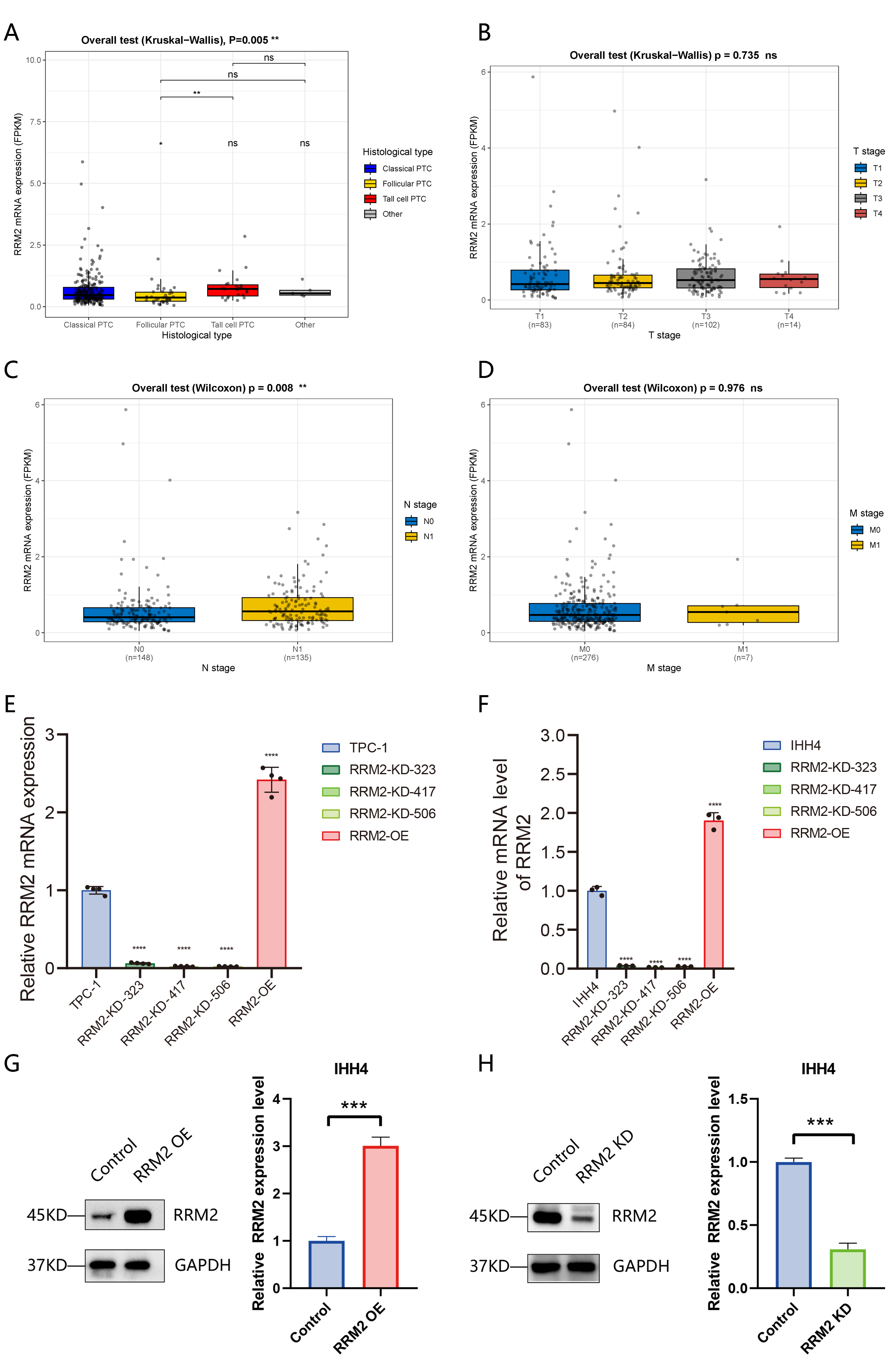

Supplement: Supplementary file 5 [file Image4.tif]

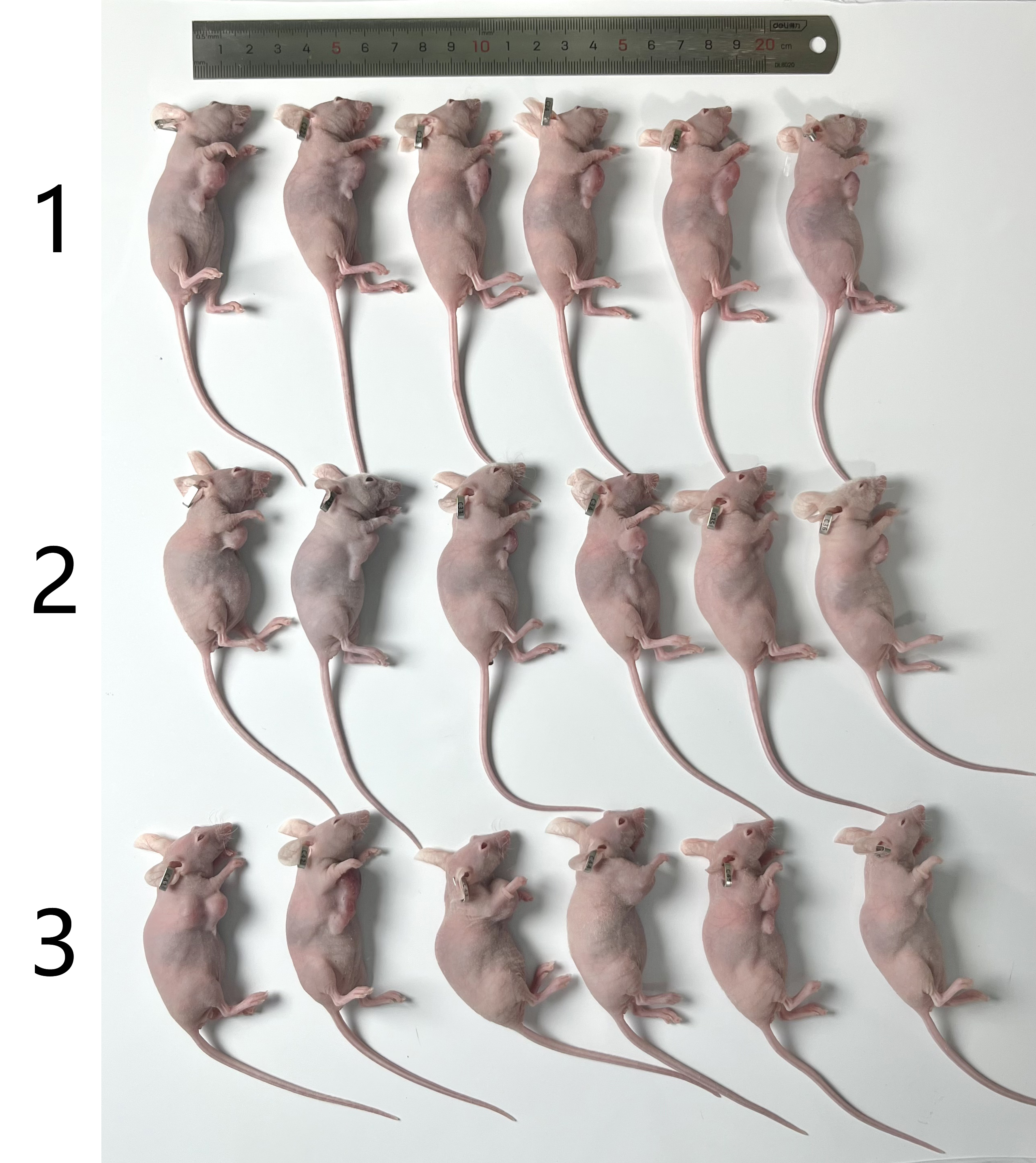

Supplement: Supplementary file 6 [file Image5.jpeg]

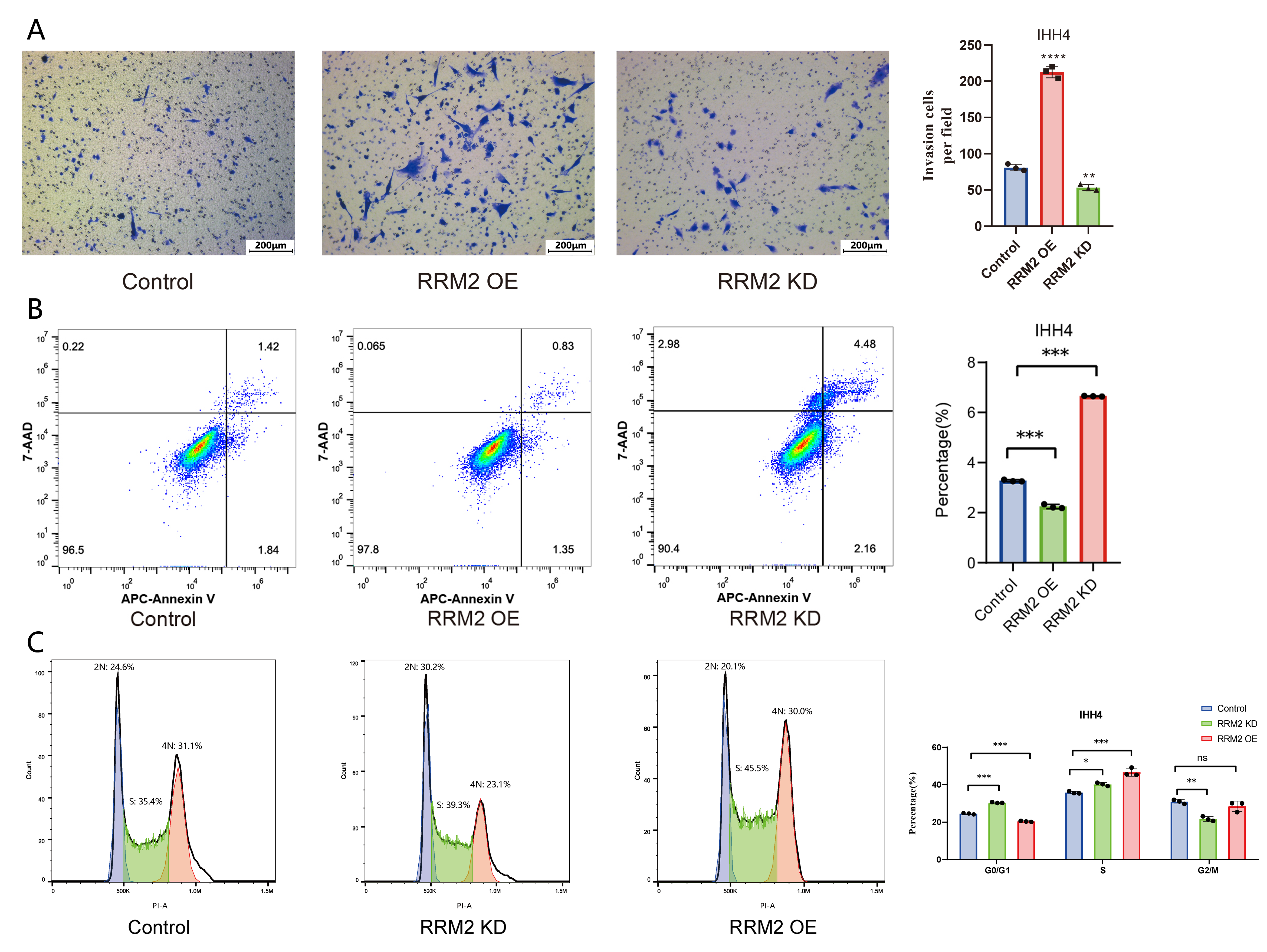

Supplement: Supplementary file 7 [file Image6.tif]

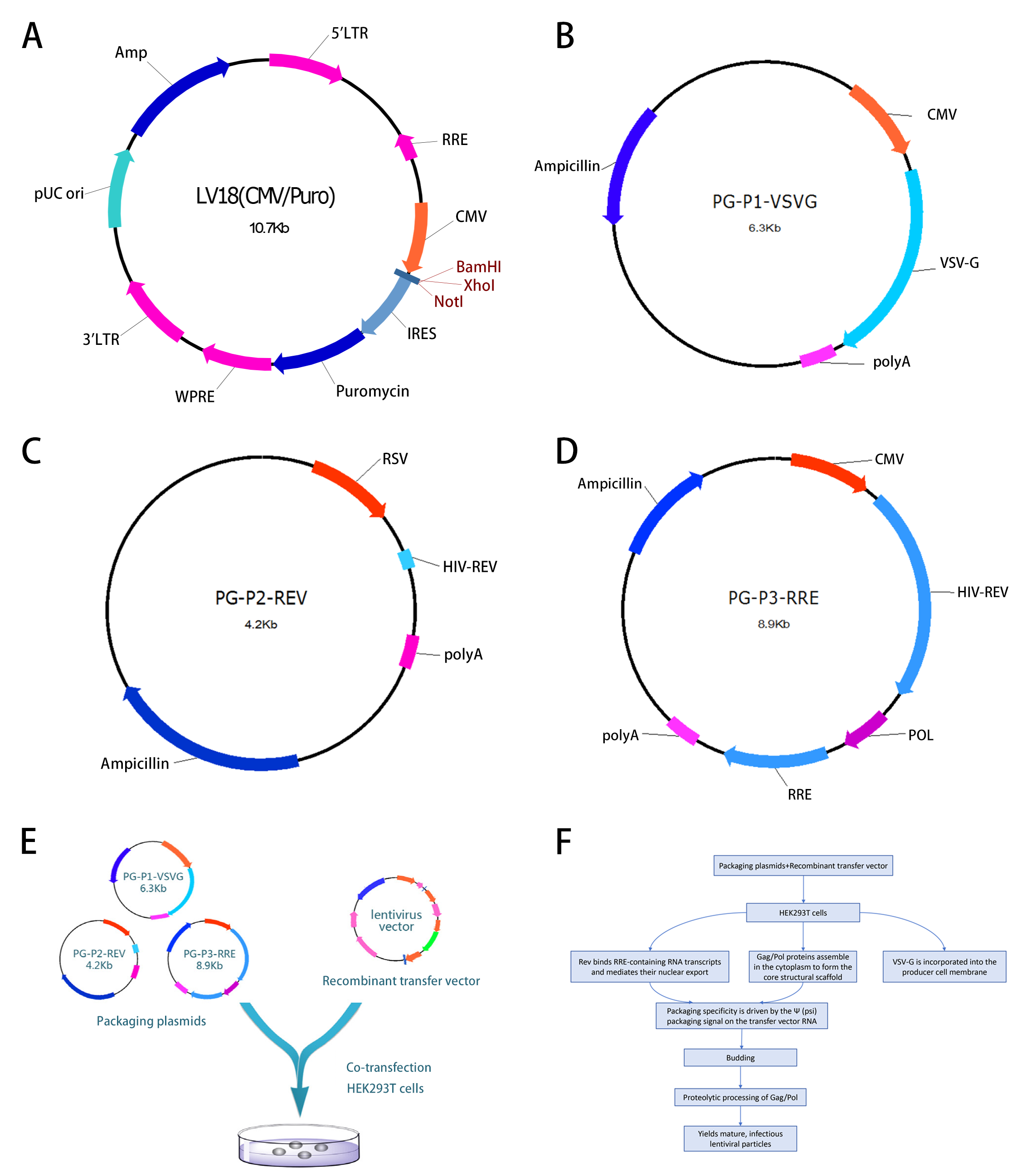

Supplement: Supplementary file 8 [file Image7.tif]
